# Hepatitis B virus core protein phosphorylation: Identification of the SRPK1 target sites and impact of their occupancy on RNA binding and capsid structure

**DOI:** 10.1371/journal.ppat.1007488

**Published:** 2018-12-19

**Authors:** Julia Heger-Stevic, Peter Zimmermann, Lauriane Lecoq, Bettina Böttcher, Michael Nassal

**Affiliations:** 1 University Hospital Freiburg, Department of Medicine II / Molecular Biology, Faculty of Medicine, University of Freiburg, Freiburg, Germany; 2 Biological Faculty, University of Freiburg, Freiburg, Germany; 3 Institut de Biologie et Chimie des Protéines, University of Lyon1, Lyon, France; 4 Department of Biochemistry, Biocenter, University of Würzburg, Würzburg, Germany; University of California, San Diego, UNITED STATES

## Abstract

Hepatitis B virus (HBV) replicates its 3 kb DNA genome through capsid-internal reverse transcription, initiated by assembly of 120 core protein (HBc) dimers around a complex of viral pregenomic (pg) RNA and polymerase. Following synthesis of relaxed circular (RC) DNA capsids can be enveloped and secreted as stable virions. Upon infection of a new cell, however, the capsid disintegrates to release the RC-DNA into the nucleus for conversion into covalently closed circular (ccc) DNA. HBc´s interactions with nucleic acids are mediated by an arginine-rich C terminal domain (CTD) with intrinsically strong non-specific RNA binding activity. Adaptation to the changing demands for nucleic acid binding during the viral life cycle is thought to involve dynamic phosphorylation / dephosphorylation events. However, neither the relevant enzymes nor their target sites in HBc are firmly established. Here we developed a bacterial coexpression system enabling access to definably phosphorylated HBc. Combining Phos-tag gel electrophoresis, mass spectrometry and mutagenesis we identified seven of the eight hydroxy amino acids in the CTD as target sites for serine-arginine rich protein kinase 1 (SRPK1); fewer sites were phosphorylated by PKA and PKC. Phosphorylation of all seven sites reduced nonspecific RNA encapsidation as drastically as deletion of the entire CTD and altered CTD surface accessibility, without major structure changes in the capsid shell. The bulk of capsids from human hepatoma cells was similarly highly, yet non-identically, phosphorylated as by SRPK1. While not proving SRPK1 as the infection-relevant HBc kinase the data suggest a mechanism whereby high-level HBc phosphorylation principally suppresses RNA binding whereas one or few strategic dephosphorylation events enable selective packaging of the pgRNA/polymerase complex. The tools developed in this study should greatly facilitate the further deciphering of the role of HBc phosphorylation in HBV infection and its evaluation as a potential new therapeutic target.

## Author summary

The liver-pathogenic hepatitis B virus (HBV) is a small enveloped DNA virus that replicates through reverse transcription of a pregenomic (pg)RNA. This requires specific encapsidation of pgRNA and viral polymerase into a shell of 240 core protein (HBc) subunits. Capsid-internal formation of relaxed circular (RC) DNA enables the particle to leave the cell as stable virion; yet, when infecting a new cell it must release the RC-DNA for conversion into another, plasmid-like DNA that templates new viral RNAs. This up and down in nucleic acid interactions is presumably regulated by transient phosphorylation of HBc, mainly in its arginine-rich C terminal domain (CTD) which displays strong non-sequence-specific RNA binding. However, neither the phosphorylation sites nor the relevant enzymes are well defined. We developed a recombinant system to produce kinase-specific phospho-HBc species, and adapted a feasible gel assay for their separation. By mutagenesis and mass spectrometry we identified seven target sites for a major candidate kinase, SRPK1, in the CTD. As full SRPK1 phosphorylation thwarted non-specific RNA binding the comparably high phosphorylation of HBc in human cells suggests how specific pgRNA encapsidation might be achieved. Our new tool set will facilitate disentangling the role of HBc phosphorylation in HBV infection and exploiting it as potential therapeutic target.

## Introduction

Chronic infection with hepatitis B virus (HBV) puts more than 250 million people at a greatly increased risk to develop terminal liver disease [1]. HBV, the prototypic hepadnavirus, is a small enveloped virus that replicates its 3 kb DNA genome through capsid-internal reverse transcription of a pregenomic (pg) RNA (reviewed in [2, 3]). The virion envelope consists of a lipid bilayer into which the small (S), middle (M; PreS2/S) and large (L; PreS1/PreS2/S) surface proteins are embedded [4–6]. Binding of L to the HBV receptor sodium taurocholate cotransporting polypeptide (NTCP) is essential for infection (reviewed in [7]); in addition, L contributes a "matrix domain" that interacts with the capsid for virion morphogenesis (reviewed in [8]).

**Fig 1:**
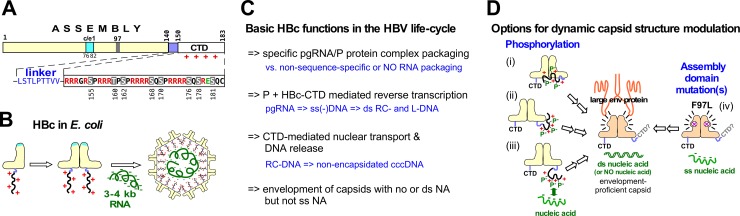
Structural and functional aspects of HBc. **(A) Domain organization.** Numbers refer to aa positions. The immunodominant c/e1 epitope, in the 3D structure at the tips of the capsid spikes, and mutation F97L are indicated. Residues 141–149 (blue) link the assembly domain to the CTD which encompasses 16 basic R residues (red) and one acidic E residue (green). Phosphorylation of the S/T residues would reduce positive charge. **(B) Non-sequence-specific RNA packaging by HBc in E. coli.** HBc forms dimers that spontaneously assemble into CLPs. With HBc183, the basic CTDs mediate encapsidation of ~3–4 kb non-sequence-specific RNA. In HBV infection this would compete with specific encapsidation of viral pgRNA. **(C) Basic functions of HBc in the HBV life-cycle.** HBc is required to interact with different forms of viral genomic nucleic acid (NA) while avoiding interactions with irrelevant NAs. Progeny virus particles leaving a cell must be environmentally stable, yet upon infection of a new cell they must orderly release the viral NA for nuclear cccDNA formation. This implies temporal changes in NA binding capacity and capsid structure, including CTD disposition. **(D) Options for capsid structure modulation.** Regulated structure modulation is manifest by the selective, L protein dependent envelopment of dsDNA (or no NA) containing capsids (symbolized by the altered shape and color of the HBc dimer in the center) but not of capsids harboring RNA or ssDNA. Sensing the type of internal NA must involve the CTDs, and likely their phosphorylation status. The negatively charged phosphoryl groups could directly affect CTD—assembly domain interactions (i) and/or CTD disposition (ii), or act indirectly through altered NA binding (iii). Mutations like F97L evade this dependency, possibly by *a priori* promoting an envelopment-proficient structure.

The icosahedral HBV capsid (core particle) is composed of 120 dimers (triangulation number T = 4) of a single core protein (HBc) species of 183–185 amino acids (aa) in length; a minor capsid class (T = 3) comprises 90 HBc dimers. The HBc monomer encompasses an N terminal assembly domain [9], linked through residues 141–149 [10] to an arginine-rich C terminal domain (CTD; Fig 1A). The CTD is crucial for specific co-encapsidation of a complex of pgRNA and viral polymerase (P protein) during replication but it can also mediate non-sequence-specific packaging of RNA (Fig 1B), e.g. when HBc is expressed in *E*. *coli* [9, 11–13]. Structural analyses of such capsid-like particles (CLPs), mostly from CTD-less variants like HBc149 [9], revealed five α-helices [14–16]. Helices α3 and α4 form an antiparallel hairpin; for dimerization, two such hairpins associate into four-helix bundles which protrude as spikes from the capsid surface (Fig 1B). Helix α5 plus the downstream sequence to position 140 harbor the major inter-dimer contacts.

Little is known about the structure of the CTD; in current CLP structures the visible sequence commonly ends within the linker [10, 14–19]. Regarding CTD disposition most data support a luminal localization [20–23]. However, a permanent internal disposition as well as a static nucleic binding capacity are incompatible with the full set of HBc functions in the viral life-cycle (Fig 1C). Beyond pgRNA/P protein encapsidation these include CTD-mediated reverse transcription [24] into single-stranded (ss) minus-DNA and then partly double-stranded (ds) relaxed circular (RC) DNA [24–26]; capsid envelopment for secretion of virions [8]; and, upon infection of a new cell, transport of the viral genome to the nuclear pore so as to release the RC-DNA into the nucleoplasm [27, 28] for conversion into covalently closed circular (ccc) DNA [29, 30]. Nuclear transport requires binding of CTD-encoded nuclear localization signals (NLSs; [31]) to cytosolic transport receptors [32–35] such that at least one CTD per capsid must become exposed [18, 27, 32, 36, 37].

Hence the capsid must safely stow ss and also ds nucleic acid with twice as many negative charges and then orderly let go of it.

A likely mechanism underlying these CTD dynamics is transient phosphorylation, early-on hinted at by a capsid-associated protein kinase activity [38, 39]. However, neither this kinase nor other potentially HBc-relevant kinases have unambiguously been identified. Proposed candidates include Ca^2+^ and/or lipid-activated protein kinase C (PKC; [36, 40]); cyclic AMP dependent protein kinase A (PKA; [41, 42]); serine/arginine-rich protein kinase 1 and 2 (SRPK1, SRPK2; [43]); cyclin-dependent kinase 2 (CDK2; [44]); and polo-like kinase 1 (PLK1; [45]). Major analytical challenges are the presence in human cells of >500 protein kinases plus >200 phosphatases [46]; the repetitve nature of the HBc CTD sequence (Fig 1A); the limited specificity of pharmacological kinase inhibitors; and especially the lack of assays that feasibly distinguish non-phosphorylated and differently phosphorylated HBc species.

As a surrogate, various studies assessed the impact of serine and/or threonine (S/T) replacements in HBc by alanine (A) to prevent phosphorylation, and by aspartic acid (D) or glutamic acid (E) to mimic phosphorylation [47–50]. Collectively these data suggest that phosphorylation is necessary early and some dephosphorylation late during replication. However, genetic mimics cannot model the dynamics of phosphorylation. Phospho-HBc-specific antibodies [51, 52] are valuable but they interrogate only few of the many options for phosphorylation site occupancy. Correlation with specific replication states is further convoluted by the variety of capsid forms, including nonenveloped capsids [53] and the recently found enveloped genome-less capsids ("empty virions"; [52]) which seem to by far outnumber infectious virions (reviewed in [54]).

The most direct evidence for the dynamics in core protein phosphorylation comes from duck HBV (DHBV). Intracellular DHBV core protein (DHBc) showed up to four distinct phosphorylation-dependent bands in normal SDS-PAGE [55] whereas virion-derived DHBc displayed a single, non-phosphorylated band, as confirmed by mass spectrometry (MS) [56]. The presence in virions of mostly mature viral ds DNA [57], similarly observed for HBV [58], supported the "maturation signal" hypothesis [57] whereby capsid-internal genome maturation, perhaps sensed by the CTDs and/or their phosphorylation status, exposes interaction sites for L protein on the capsid surface (Fig 1D). The finding that also empty though not ss nucleic acid containing capsids can be enveloped prompted a revised model whereby RNA or ssDNA actively inhibit envelopment [52, 59]. A cryoEM comparison between recombinant RNA containing capsids and serum virus-derived, supposedly dsDNA bearing capsids had indeed revealed subtle differences [17]. However, at the time empty virions in serum [52, 60] were not known; hence potential envelopment-relevant structural differences and their correlation with HBc phosphorylation remain open (Fig 1C, i-iii).

Some natural HBc mutations such as F97L [61–63] promote secretion of immature, ssDNA containing virions. Such capsid proteins might by default adopt an envelopment-proficient conformation (Fig 1D). Yet, while affecting in vitro assembly [64] no differences in stability or morphology between wild-type and F97L HBc particles were detectable [63, 65]. However, a potential role of phosphorylation could not be assessed at the time.

In sum a large body of data supports correlations between HBc phosphorylation, genome maturation and capsid structure, including CTD disposition and envelopment-competence; however, fundamental information for disentangling these interdependencies is lacking.

In order to generate such information we here established the efficient recombinant production of distinct phospho-HBc species and showed the feasibility of Phos-tag SDS-PAGE [66] for their differentiation. Using MS and mutagenesis we identified seven hydroxy amino acid residues in the CTD as target sites for SRPK1 [34, 43] while PKA and PKC modified fewer, yet to be mapped sites. Full occupancy of the SRPK1 sites massively reduced HBc183 CLP RNA content while abrogation of one phospho-site restored substantial RNA binding. Full phosphorylation also caused differences in CTD accessibility to trypsin, regardless of the F97L mutation. Importantly, in human hepatoma cells the bulk of HBc appeared as highly phosphorylated as SRPK1-coexpressed HBc. These data pave the way for a comprehensive characterization of the substrate properties of hepadnaviral core proteins for individual kinases and phosphatases. Virologically they suggest a mechanism whereby high-level HBc phosphorylation principally suppresses RNA binding whereas one or few dephosphorylation events could enable specific packaging of the pgRNA/P protein complex.

## Results

### A robust bacterial coexpression system for phosphorylated HBV core protein

Eukaryote-like S/T protein kinases are rare in eubacteria [67] and apparently absent from *E*. *coli* laboratory strains. Hence co-expression of HBc with a mammalian kinase should allow the background-free evaluation of its action on HBc. In a first step we created T7 promoter-based vectors carrying an *E*. *coli* codon usage adapted HBc gene (HBc183opt) to boost expression of full-length HBc183 (Fig 1A). CLP yields were indeed around three-fold higher (S1 Fig) compared to the older non-optimized HBc gene [68] in the same pET28a2 vector [69, 70], and they increased further using pRSF-Duet derived vectors (see below).

For the coexpression approach the recombinant kinase must be enzymatically active and target sites in HBc must not be sequestered by CLP formation. We thus chose human SRPK1 as the first candidate HBc kinase. Beyond its very high affinity for the HBc CTD [34, 43, 71] we had already shown HBc phosphorylating activity of the full-length enzyme in bacteria [11]; here we used an N terminally His6-tagged variant, NHisSRPK1ΔNS1, in which an internal deletion boosts soluble expression in bacteria [72].

To minimize experimental variations seen when HBc and kinase were expressed from two separate plasmids [11] we devised a single-vector dual expression system; it features a Tet repressor / anhydrotetracycline (AHT) inducible Tet promoter cassette plus an isopropyl β-D-1-thiogalactopyranoside (IPTG) regulatable T7 promoter cassette (S2 Fig). Functionality was shown by inducer-specific expression of eGFP from one and mCherry from the other cassette (S2 Fig). HBc183opt in the T7 cassette was expressed at up to five-fold higher levels than from the pET28a2 vector (S3 Fig). Of note, expression from the Tet cassette was affected by the specific ORF sequence. For instance, the basal level of NHisSRPK1ΔNS1 expression without AHT was still about one third as high as with inducer (S3A Fig); hence in subsequent SRPK1 coexpression experiments AHT was omitted.

Either way, in line with its high affinity for HBc [34, 43] most of the SRPK1 cosedimented with the CLPs, both from wild-type and F97L HBc (S3 Fig); separation was achieved by immobilized metal ion chromatography (IMAC) under semi-denaturing conditions (S4 Fig). Altogether, the system provided easy access to milligram amounts of CLPs per 100 ml of induced bacterial culture.

### Strongly reduced RNA content of HBc183 CLPs by SRPK1 coexpression

Low level co-expression in *E*. *coli* of full-length SRPK1 with HBc183 had generated a mixture of HBc species containing from one to several phosphoryl groups [11]; this prompted the follow-up use of a triple S>E variant, HBc183-EEE (S155E_S162E_S170E) as a homogeneous genetic mimic of such partially phosphorylated HBc183 [34, 35]. HBc183-EEE CLPs contained ~25% less RNA than wild-type HBc183 CLPs [73]. To evaluate the impact on CLP RNA content of the much higher NHisSRPK1ΔNS1 expression in our new system we separated the CLPs by NAGE, then stained packaged RNA by ethidium bromide (EB) and the protein shell by Coomassie Brillant Blue (CB) [9, 74]; other stains, e.g. Sybr Green 2 (SG2) for RNA and Sypro Ruby (SR) for protein [75], could be used instead (see below). The intensity of RNA vs. protein stain per band is proportional to the CLP´s RNA content.

**Fig 2:**
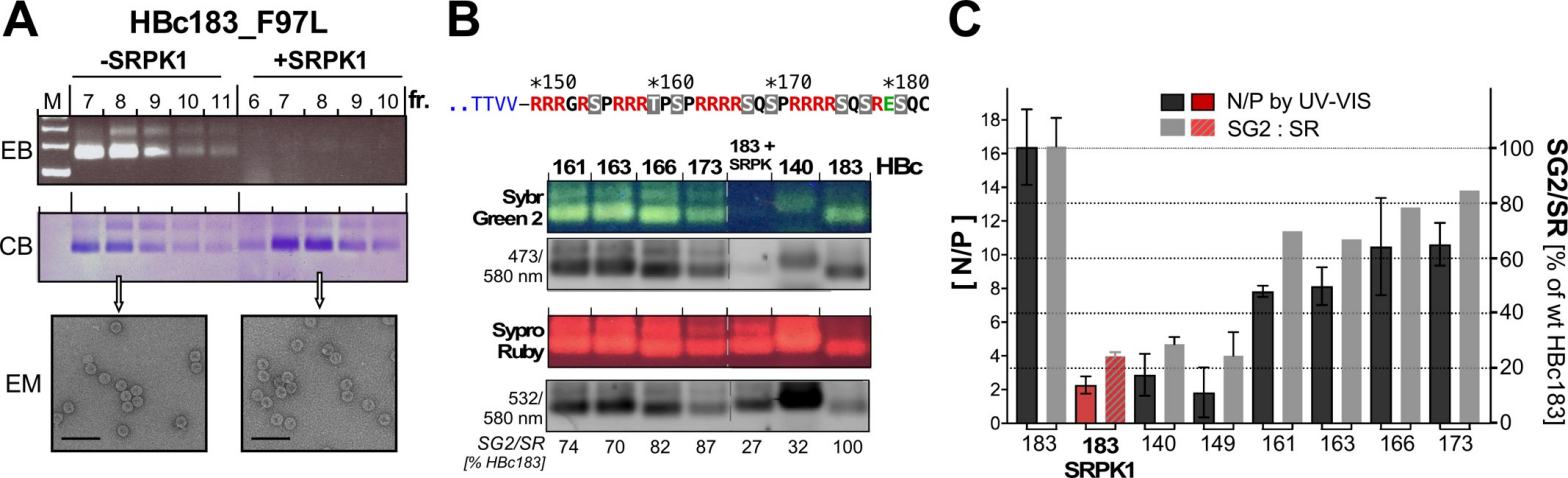
Coexpression with SRPK1 strongly reduces HBc CLP RNA content. **(A) Visualization of RNA content in HBc183_F97L CLPs by NAGE and RNA vs. protein staining.** HBc183_F97L CLPs expressed in the absence (-) or presence (+) of NHisSRPK1ΔNS1 were enriched to sucrose gradient sedimentation. The indicated fractions were separated by NAGE in the presence of ethidium bromide (EB); subsequently proteins were stained by Coomassie Blue (CB). Note the high vs. low EB to CB signal ratios in the -SRPK1 vs. the +SRPK1 samples but their nearly identical mobility. Negative staining EM (EM) did not reveal differences between the two HBc183_F97L samples, nor between the respective wild-type HBc183 samples (S3B Fig). **(B) Impact of NHisSRPK1ΔNS1 coexpression vs. CTD truncations on RNA content.** CLPs from the indicated truncated HBc variants were subjected to NAGE in EB-free gels, then stained with Sybr Green 2 (SG2) for RNA; after documentation the gels were counterstained with Sypro Ruby (SR) for protein. Green and red signals were semiquantitatively evaluated by laser scanning at the indicated conditions (grey-scale panels). The ratios of SG2 to SR fluorescence in each sample are given as percent of the respective value for HBc183 CLPs expressed without kinase. **(C) Similarly strong reduction in absolute CLP RNA content by NHisSRPK1ΔNS1 coexpression as by CTD deletion.** RNA contents of the indicated HBc CLPs (in nt per HBc protein monomer (N/P)) were calculated from UV/VIS spectra [73]. Black bars show the mean N/P values ± SD (n≥3). For comparison, the relative values derived from (B) are shown as grey bars; the scale was set such that the 100% value (HBc183) matched the mean N/P value (~16) of the same sample. In either assay, the RNA content of HBc183 and HBc183_F97L CLPs coexpressed with SRPK1 was as low as that of HBc140 CLPs.

Fig 2A shows such an analysis for HBc183_F97L expressed in the absence (-SRPK) vs. presence of SRPK1. Despite much weaker EB staining the +SRPK1 samples showed even stronger CB staining; in either case abundant CLPs were visible in negative staining EM; wild-type HBc183 CLPs gave comparable results. To appreciate the extent of reduction in RNA content caused by NHisSRPK1ΔNS1 coexpression we used a reference set of CLPs from C terminally truncated HBc proteins, starting with the CTD-less variant HBc140. SG2 and SR allowed semiquantitative assessment of the relative fluorescence intensities of each band (Fig 2B) using a laser scanner (Typhoon FL7000, GE Healthcare). The ratio of RNA: protein fluorescence for non-phosphorylated HBc183 CLPs was set to 100%, to which the ratios for the other CLPs were normalized. Accordingly, SRPK1-coexpressed HBc183 CLPs contained nominally 27% as much RNA as the HBc183 reference CLPs, very similar to CTD-less HBc140 CLPs. For the other variants, the ratios increased with increasing CTD length, in accord with the electrostatic "charge balance" hypothesis [13, 26, 76].

An estimate of absolute CLP RNA content as molar ratio of nucleotides (N) per core protein (P) subunit (N/P) was obtained from UV-VIS spectra by deconvoluting the superimposed RNA and protein absorbances [73]; spectra were recorded using gradient-enriched CLPs incubated with 1/100 the amount of (w/w) of RNase A and subsequently dialyzed to remove non-packaged RNA. For HBc183 CLPs we obtained (N/P) values ± standard deviation (SD) of 16.4±2.2 (n = 8); for a T = 4 CLP this corresponds to an RNA content equivalent to ~4,000 nt, similar to other reports [12, 73]. Much in contrast, SRPK1-coexpressed HBc183 CLPs gave a ratio of only 2.3±0.5 (n = 8), not significantly different from CTD-less HBc140 (2.9±1.3; n = 4) and HBc149 CLPs (1.8±1.5; n = 4). Intermediate RNA contents were found for HBc161, 163, 166 and 173 CLPs, as shown in Fig 2C in comparison with the NAGE/SG2:SR procedure. While the apparent overestimation of low RNA contents by the latter method could later be reduced by several improvements (Materials and Methods), both data sets corroborated that SRPK1 coexpression reduced RNA content as strongly as deletion of the entire CTD. This implied a phosphorylation extent sufficient to shift assembly to an RNA-independent, protein-protein interaction driven mechanism [37, 77], perhaps by neutralizing most of the positive CTD charges [13].

### Phos-tag SDS-PAGE indicates high-level HBc183 phosphorylation by SRPK1ΔNS1

**Fig 3:**
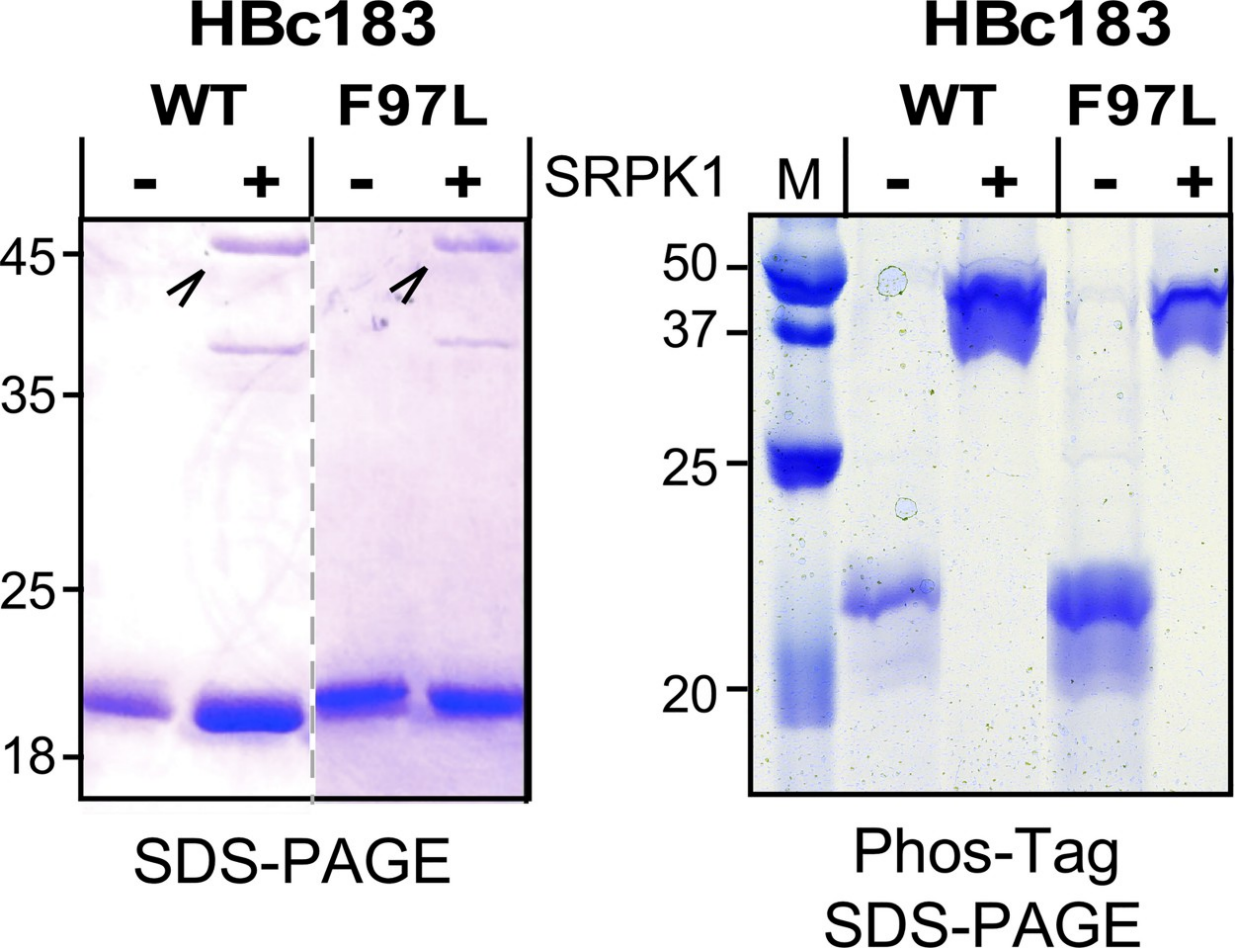
Strong and selective retardation of SRPK1-coexpressed wild-type and F97L HBc183 in Phos-tag SDS-PAGE. Samples from the indicated gradient-enriched CLPs expressed alone (-) or coexpressed with SRPK1 (+) were separated by normal SDS-PAGE (left panel) or by Mn^2+^ Phos-tag SDS-PAGE (right panel). The weak bands at the 45 kDa position marked by arrowheads (left panel) represent SRPK1 that had co-sedimented with the HBc CLPs.

The Phos-tag chelator coordinated with two Me^2+^ ions binds to phosphoryl groups, including in proteins [78]. In SDS-PAGE gels containing copolymerized Phos-tag acrylamide and loaded with Mn^2+^ or Zn^2+^ phospho-proteins migrate more slowly than their non-phosphorylated forms [66]. Hence this technique appeared attractive to detect HBc phosphorylation. After optimization we routinely used home-made gels containing 75 μM Phos-tag acrylamide loaded with 150 μM Mn^2+^. As shown in Fig 3, in normal SDS-PAGE both HBc183 and HBc183_F97L migrated to the same ~21 kDa position, regardless of SRPK1 coexpression. In Phos-tag SDS-PAGE, however, exclusively the kinase-coexpressed proteins exerted a drastically reduced mobility.

Band ladders produced in in vitro SRPK1 phosphorylation and lambda phosphatase dephosphorylation reactions with a non-assembling GFP-CTD fusion protein suggested phosphorylation at five or more positions. Analogous coexpression of HBc183 with the catalytic domains of PKA (Genbank NP_032880.1) and PKC theta (Genbank NP_001269573.1), chosen owing to their reportedly functional expression in *E*. *coli* [79, 80], caused a less pronounced retardation (see below).

### Mass spectrometry defines seven SRPK1 phosphorylation sites in HBc

The number of phosphoryl groups introduced into HBc183 by SRPK1 was analyzed by matrix-assisted laser desorption/ionization (MALDI) time-of-flight (TOF) MS. The major MH+ peaks in the relevant 21 kDa region of the spectra had mass/charge (m/z) values of 21,103 for mono-expressed HBc183 and 21,664 for SRPK1-coexpressed HBc183. These values match most closely to the calculated masses of 21,116 Da for completely unmodified HBc183, and to 21,676 Da for a seven-fold phosphorylated HBc183 (S5 Fig; each phosphoryl group contributes ~80 Da). Notably, for SRPK1-coexpressed HBc140 and HBc149 the main peaks still corresponded to the non-phosphorylated proteins; hence the assembly domain and the linker sequence lack SRPK1 target sites.

**Fig 4:**
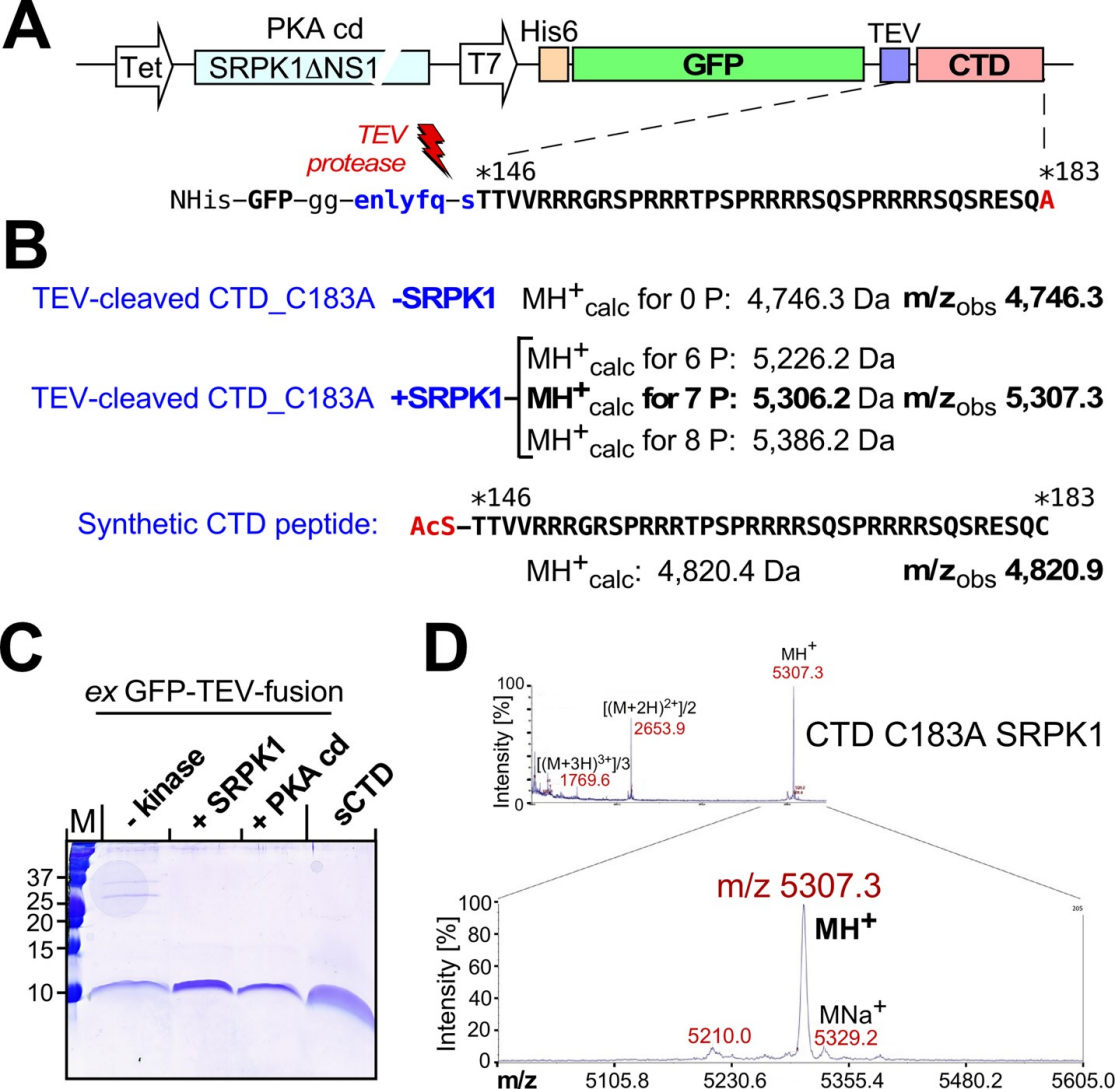
Accurate MS confirmation of seven SRPK1 phosphorylation sites in the HBc CTD. **(A) Genetic structure of the non-assembling CTD fusion constructs.** The dual promoter vectors carried an ORF for non-His-tag kinase (SRPK1ΔNS1, or the catalytic domain of PKA) plus an ORF for a His-tagged GFP protein to which the CTD of HBc variant C183A was linked via a TEV protease recognition site; see text and S1 Protocol for details. **(B) Calculated MH**^**+**^ **masses of the indicated CTD derivatives vs. observed m/z values.** Note the excellent agreement for a seven-fold phosphorylated CTD species in the +SRPK1 sample. **(C) SDS-PAGE of the purified CTD peptides from the indicated GFP fusion proteins.** The sequence of the chemically synthesized N-acetylated sCTD peptide carrying the genuine C183 residue is shown in (B). **(D) MALDI-TOF spectrum of the +SRPK1 CTD sample from (C).** Note the predominance of the m/z peak corresponding to seven-fold phosphorylated CTD. Mass spectra for the other CTD peptides are shown in S6 Fig.

For more accurate mass determination we devised a method (S1 protocol) to isolate just the CTD peptide from a tobacco etch virus (TEV) protease cleavable NHisGFP-TEV-CTD fusion protein (Fig 4A), expressed with or without kinase. Cleavage reduces the relevant masses to ~5,000 Da, with a synthetic N-acetylated CTD peptide serving as reference (Fig 4B). Methods development included replacement of the C terminal Cys residue by Ala (C183A in full-length HBc numbering). In brief, the NHisGFP-TEV-CTD protein was enriched by Ni^2+^ IMAC under semi-denaturing conditions, then incubated with His-tagged TEV protease [81] to release the CTD peptide; all His-tagged components (uncleaved GFP fusion protein, the GFP-containing cleavage product and TEV protease) were removed by another round of IMAC. The clipped-off CTD peptide in the flow-through was concentrated and further enriched by ultrafiltration. An SDS-PAGE analysis of the final products obtained upon coexpression with SRPK1ΔNS1 or the PKA catalytic domain (PKAcd) or without kinase, alongside the synthetic CTD peptide, is shown in Fig 4C.

Importantly, MS of the SRPK1 sample (Fig 4D) showed one major peak with excellent agreement to a seven-fold but not six- or eight-fold phosphorylated CTD peptide. Hence there are seven SRPK1 target sites in the HBc sequence 146–183. The m/z values for the synthetic and the non-phosphorylated fusion protein-derived CTD peptide were <1 Da off the calculated masses (S6 Fig). The PKA-coexpressed sample showed two main peaks, matching four and five phosphoryl groups, respectively; for full-length HBc183 as substrate the products from PKA and PKC coexpression carried only three phosphoryl groups (S7 Fig), possibly due to assembly-mediated target site sequestration.

### Mapping of the seven SRPK1 phosphorylation sites by mutagenesis plus MS

**Fig 5:**
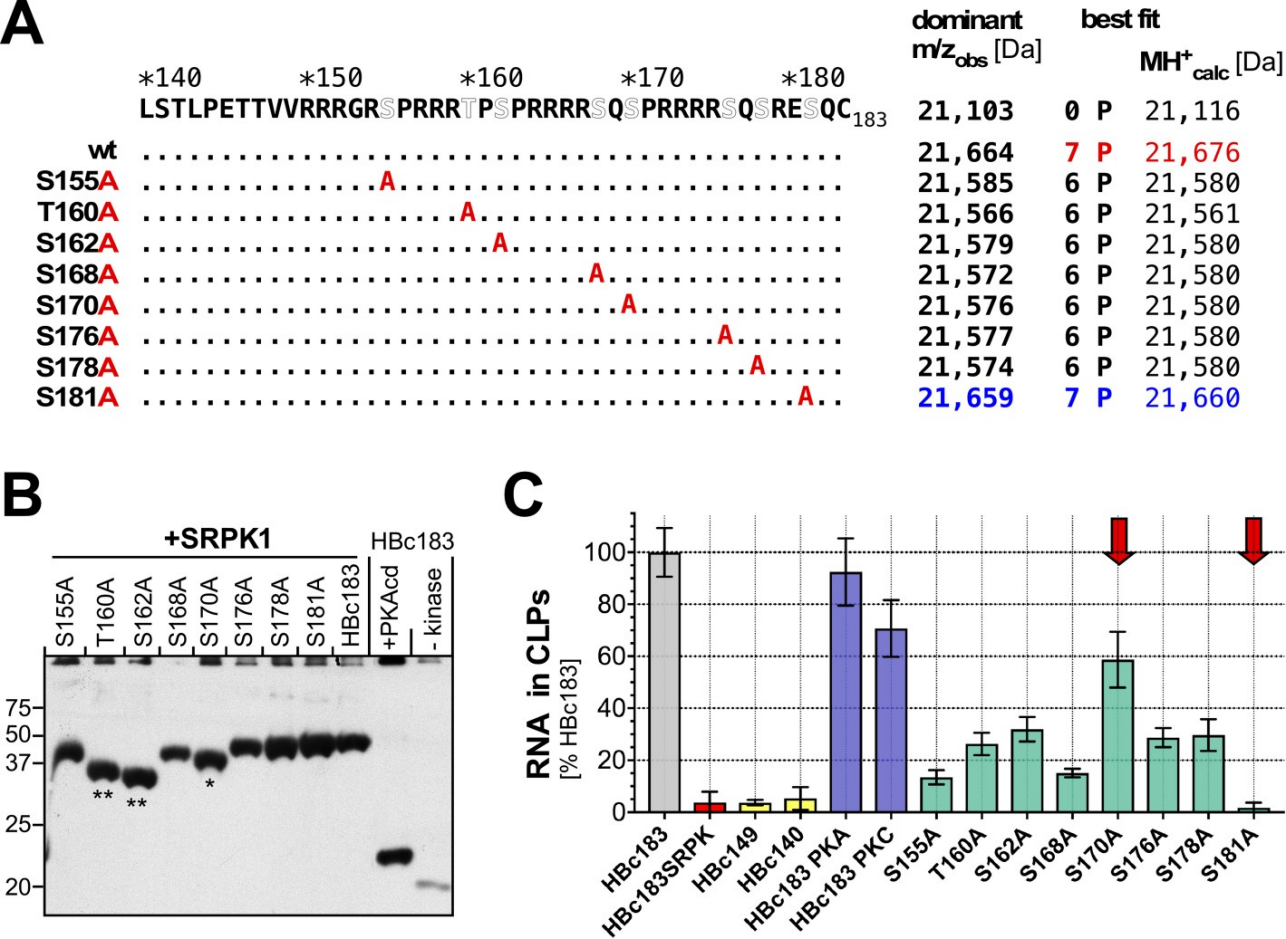
Mapping the SRPK1 phosphorylation sites in the HBc CTD by mutation and MS. **(A) Position of S/T>A mutations, dominant m/z peak observed and calculated MH+ mass of best-fitting phospho-species.** Note that S181A is the only single-site variant with a best-fit m/z for seven-fold phosphorylation; all other single-site mutants showed the best match to six phosphoryl groups. **(B) Impact of number and position of phospho-sites on Phos-tag SDS-PAGE mobility.** Following Phos-tag SDS-PAGE HBc proteins were analyzed by immunoblotting using the HBc assembly-domain specific mAb 1D8. All SRPK1-coexpressed proteins contained seven (HBc183, S181A) or six phosphoryl groups (all others) but variants S170A (*) and especially T160A and S162A (**) were less retarded, even though much more than three-fold PKAcd phosphorylated HB183. The impact of the mutations on recognition by the phospho-CTD specific mAb T2212 is shown in S12 Fig. **(C) Impact on CLP RNA content.** CLPs from the indicated HBc proteins were analyzed by NAGE and sequential SG2 vs. SR staining using an improved, background-reducing protocol (Materials and Methods). SG2: SR ratios are indicated in percent of that in non-phosphorylated HBc183 CLPs. Bars show the mean of ≥3 determinations; error bars represent standard deviation (SD). Original NAGE fluorograms plus data for additional variants are shown in S8 Fig. Note the exceptionally low SG2 to SR ratio for S181A and the higher ratio for S170A vs. all other single S/T>A variants (red arrows). The minor impact of PKA and PKC coexpression on RNA content (blue bars) correlated with the predominance of only three-fold phosphorylated species (S7 Fig) and their less pronounced retardation in Phos-tag SDS-PAGE (Fig 5B and Fig 9C).

To identify the seven SRPK1 sites in HBc we individually mutated the seven S residues plus one T residue in the CTD (S155, T160, S162, S168, S170, S172, S178 and S181) to A, coexpressed the mutant proteins with SRPK1 and monitored phosphorylation by MS (Fig 5A) and by immunoblotting after Phos-tag SDS-PAGE.

For all mutants but one MS revealed a best match to the six-fold phosphorylated forms, i.e. loss of one phosphorylation site. The exception was mutant S181A which still matched best to a seven-fold phosphorylated species. Hence all hydroxy amino acids in the HBc CTD including T160 yet except S181 are substrates for SRPK1. Double and triple S/T>A mutants confirmed the data as the number of lost phosphorylation sites always corresponded to the number of S/T replacements by A (or by G or R), except when S181 was mutated (S7 Fig).

In line with their at least six-fold phosphorylation all single S/T variants were similarly strongly retarded in Phos-tag SDS-PAGE as SRPK1-coexpressed wild-type HBc183, as shown by immunoblotting (Fig 5B) with the assembly domain-directed anti-HBc mAb 1D8 [82]. Notably, though, the correlation between the number of phosphorylation sites and mobility was not strictly linear. The six-fold phosphorylated variants S155A, S176A, and S178A migrated about as slowly as the seven-fold phosphorylated wild-type protein and variant S181A. In contrast, the likewise six-fold phosphorylated variants T160A, S162A and S170 (marked with asterisks in Fig 5B) had distinctly higher mobilities. Hence beyond the number of phosphoryl groups also their sequence context contributes to the interaction with the Phos-tag. Knowledge of the S/T>A variants´ phosphorylation status also enabled a more detailed characterization of the epitope requirements of mAb T2212 (see below).

We then evaluated the impact of the S/T>A mutations on CLP RNA content by the NAGE based SG2/SR staining assay (Fig 5C, S8 Fig) but using a more elaborate protocol (Material and Methods). Owing to the lower background signals the relative RNA contents of HBc140, HBc149 and SRPK1-coexpressed HBc183 as well as HBc183_S181A CLPs were now estimated to ≤10% of that in unmodified HBc183 CLPs (Fig 5C). The mostly three-fold phosphorylation by PKA and PKC caused only minor reductions (significant only for PKC). Remarkably, preventing phosphorylation at one of the seven SRPK1 sites was sufficient to increase RNA contents to generally ~20–30% of non-phosphorylated HBc183 CLPs; additional phosphorylation site mutations further increased RNA content (S8 Fig). Intriguingly, the single S170A mutation restored RNA content to ~50% of unmodified wild-type HBc183 CLPs, and this difference to the other single variants was significant (p<0.001; n = 6). Hence phosphorylation of S170 may be particularly effective in suppressing RNA binding by the HBc CTD.

### Little impact of high phosphorylation status, low RNA content and F97L mutation on CLP stability against SDS

**Fig 6:**
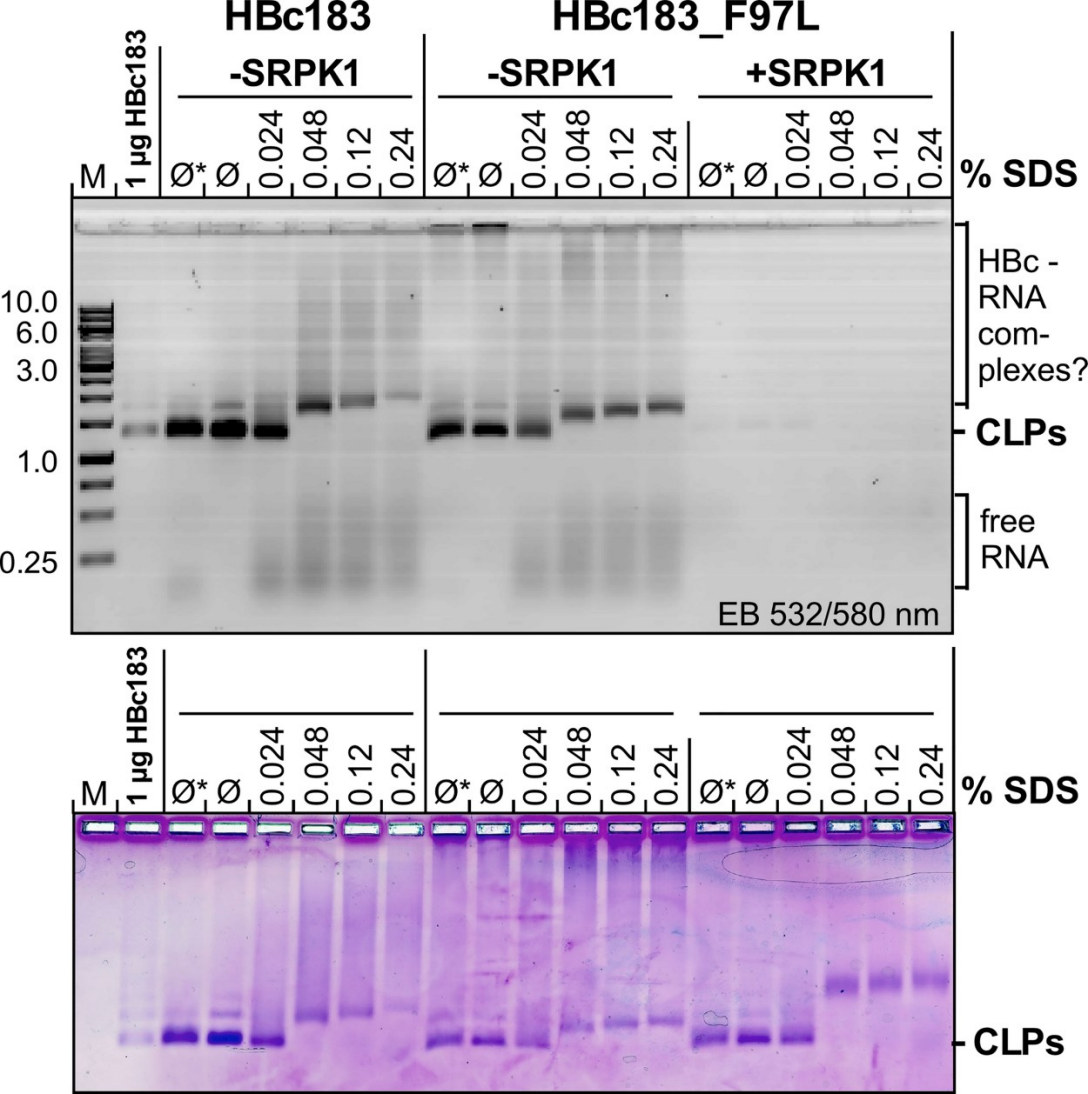
Little impact on SDS sensitivity of CLPs by high phosphorylation, low RNA content and F97L mutation. About 5 μg HBc protein from the indicated CLP preparations were directly loaded (Ø), or after 30 min incubation in non-denaturing 6x DNA loading buffer (Ø*) or in SDS-containing DNA loading buffer (NEB Purple) at the indicated final SDS concentrations, then analyzed by NAGE. A 1 kb DNA ladder (M) plus 1 μg of untreated HBc183 CLPs served as markers. EB fluorescence signals (top) were recorded using a laser scanner (excitation 532 nm/O580 nm filter). Proteins were subsequently stained by CB. In all samples the intact CLP bands became fuzzier at 0.024% SDS compared to the untreated controls, and a distinct upward mobility shift occurred at 0.048% SDS. For the non-phosphorylated CLPs (-SRPK1) this was accompanied by visible release of RNA.

HBc183 CLPs are sensitive against SDS [9, 65]; hence titration with SDS might reveal whether high-level phosphorylation and/or the concomitant low RNA content affect CLP stability. To this end we incubated HBc183 CLPs and HBc183_F97L CLPs expressed in the absence vs. presence of SRPK1 with either 1x TAE electrophoresis buffer (Ø), or a native glycerol-based DNA loading buffer (Ø*), or increasing amounts of a 6x DNA loading buffer (NEB Purple) containing 0.48% SDS (0.08% at 1x concentration). RNA and protein were monitored by EB and CB staining; expectedly, SRPK1-coexpressed CLPs showed only faint EB versus CB signals (Fig 6, right panels).

At 0.024% SDS the nonphosphorylated CLPs released some of the RNA (faster migrating EB smear) although the major band remained at the original position. At 0.048% SDS, the fast RNA smear increased, while the original CLP band disappeared in favor of a slower, less intense band from which EB signals emanated upwards to the cathode; still higher SDS concentrations enhanced these effects. CB staining of the latter bands suggests they may represent complexes of HBc183 proteins with exposed CTDs (forced to move towards the cathode) to which some of the initially encapsidated RNA is bound. In accord with previous data [65] no clear difference was detectable for wild-type vs. F97L CLPs. Interestingly the SRPK1-phosphorylated low RNA CLPs also showed a sharp mobility transition between 0.024% and 0.048% SDS, although the new products migrated a bit slower than those from the nonphosphorylated CLPs. In sum, SDS sensitivity of the HBc CLPs was neither affected by seven-fold phosphorylation of the CTD, nor by RNA content or the F97L mutation.

### Altered HBc CLP trypsin sensitivity by high-level phosphorylation and low RNA content

The high arginine content makes the entire HBc CTD an excellent substrate for trypsin; however, protease accessibility is affected by the CLP structure [37, 65, 83]. In our hands, trypsin had converted part of the unmodified wild-type HBc183 into a major product with HBc149-like mobility [83]. To reveal potential alterations of this pattern by phosphorylation, RNA content and/or the F97L mutation, we monitored the kinetics of trypsin action on non-phosphorylated vs. phosphorylated CLPs from wild-type and F97L HBc183 protein.

**Fig 7:**
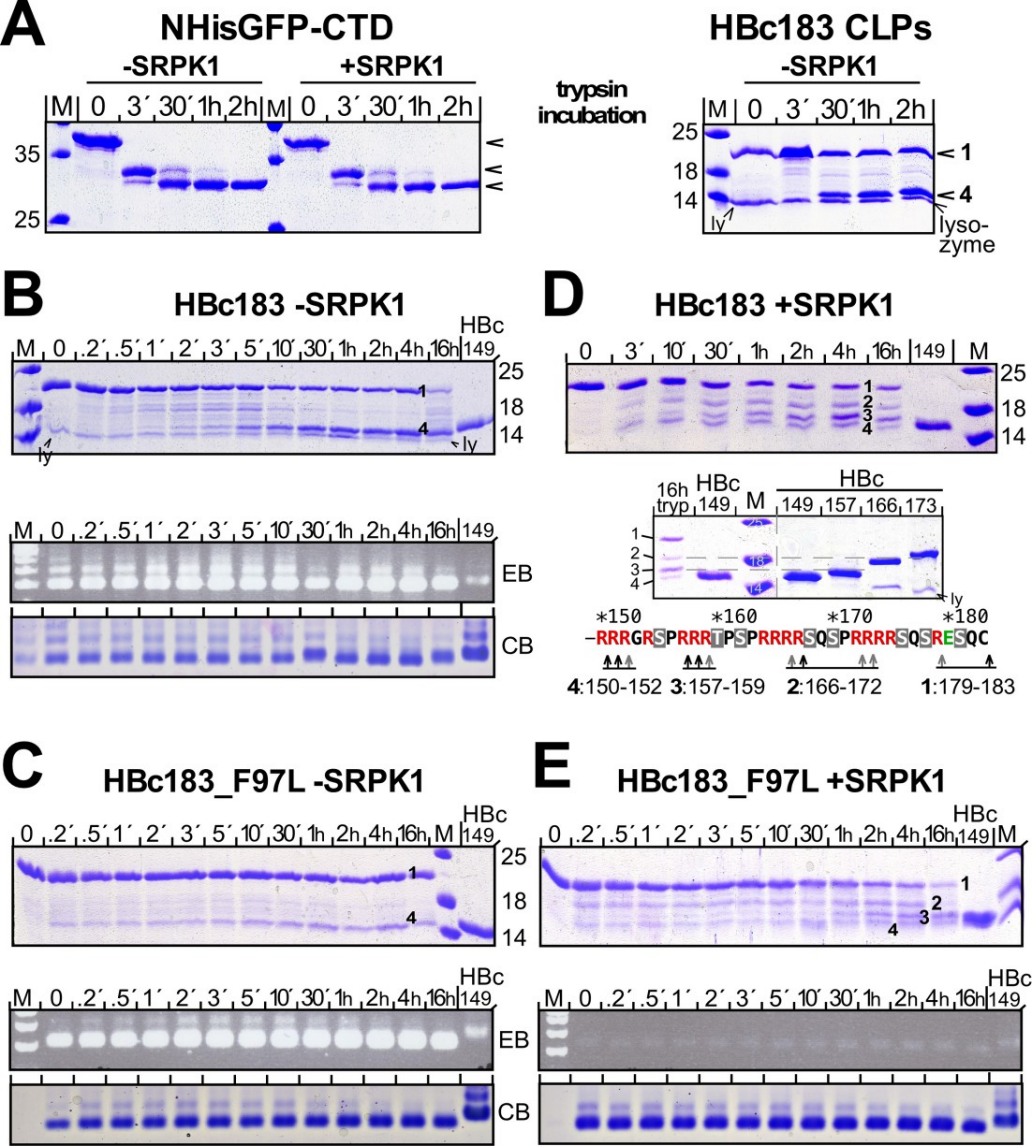
Strong impact on CLP trypsin sensitivity by high level phosphorylation and low RNA content but not the F97L mutation. **(A) Full cleavage of accessible but not CLP-borne CTD regardless of phosphorylation.** NHis-GFP-CTD fusion protein expressed with or without SRPK1, and HBc183 CLPs were incubated as indicated with 1% (w/v) trypsin; digestion was stopped by AEBSF and reactions were analyzed by SDS-PAGE and CB staining. Non-phosphorylated and phosphorylated GFP-CTD fusion protein were completely cleaved with comparable kinetics. In the CLPs less than half the HBc183 subunits were rapidly cleaved into a 15 kDa product; the 14 kDa lysozyme band (ly) in some samples originates from the cell lysis procedure. **(B) Non-phosphorylated HBc183 CLPs.** Trypsin incubation and inhibition were done as in (A). Reaction products were analyzed by SDS-PAGE and NAGE (lower panels) with EB and CB staining. Note the overall unaltered NAGE signals despite cleavage of about half the subunits; intact versus cleaved chains are labeled 1 and 4. **(C) Non-phosphorylated HBc183_F97L CLPs.** As for HBc183 CLPs, uncleaved chains (1) plus a 15 kDa band (4) were the major products (top panel); NAGE showed unaltered RNA content and CLP mobility (bottom). **(D) SRPK1-phosphorylated HBc183 CLPs.** Note the additional intermediate mobility bands 2 and 3 (top panel). Running the 16 h sample along a set of C terminally truncated HBc proteins (lower panel) allowed to approximately map the cleavage sites, as outlined in the scheme at the bottom. **(E) SRPK1-phosphorylated HBc183_F97L CLPs.** The cleavage pattern with bands 1, 2, 3, 4 and accumulation of band 3 (top panel) was as for SRPK1-coexpressed HBc183; also in NAGE (bottom panels) the much weaker RNA signals as well as the equally strong protein signals remained unaltered.

First we addressed a potential inhibition of trypsin cleavage by CTD phosphorylation by comparing the trypsin sensitivity of the non-assembling NHis-GFP-TEV-CTD fusion protein (see Fig 4A) expressed in the absence vs. presence of SRPK1. Both proteins were completely cleaved with similar kinetics (Fig 7A, left panel). Under the same conditions, HBc183 in non-phosphorylated CLPs was only partially cleaved within 30 min (Fig 7A, right panel), with about half the molecules remaining intact and the other half displaying HBc149-like mobility; this pattern remained stable over 2 h.

Higher temporal resolution (Fig 7B) revealed the transitory formation of intermediate mobility bands which had mostly disappeared at 30 min in favor of the stable HBc149-like product (labeled "4"); thereafter the ratio between presumably intact HBc183 (labeled "1") and product "4" remained constant for at least 4 h. NAGE did not reveal significant alterations in EB vs. CB staining over time (Fig 7B, lower panels). Hence loss of half the CTDs affected neither RNA content nor surface charge, indicating the overall CLP structure remained intact. Comparable results with HBc183_F97L CLPs (Fig 7C) ruled out major effects of the F97L mutation.

In contrast, SRPK1-coexpressed HBc183 CLPs (Fig 7D, top panel) presented two additional discrete, stable products ("2" and "3") of which product 3 eventually reached similar levels as the remaining full-length protein. By comparison with HBc marker proteins (Fig 7D, lower panel) products 3 and 2 resulted from cleavage between positions 157–159 and 166–172, respectively. HBc_F97L CLPs produced a very similar four-band pattern in SDS-PAGE (Fig 7E, top panel); in NAGE CLP mobility and RNA content again remained unaltered (Fig 7E, bottom panels). While quantitation of the band 1 to 4 proportions could be disturbed by the presence of not fully intact CLPs, formation of bands 2 and 3 exclusively from the phospho-CLPs strongly implies distinct steric constraints for the band 2 and 3 processing sites compared to unmodified CLPs. Scenarios compatible with the data (complete CTD extrusion vs. looping out of CTD parts) are shown in S9 Fig.

### Structural comparison of non-phosphorylated versus seven-fold phosphorylated HBc183 CLPs by cryoEM

**Fig 8:**
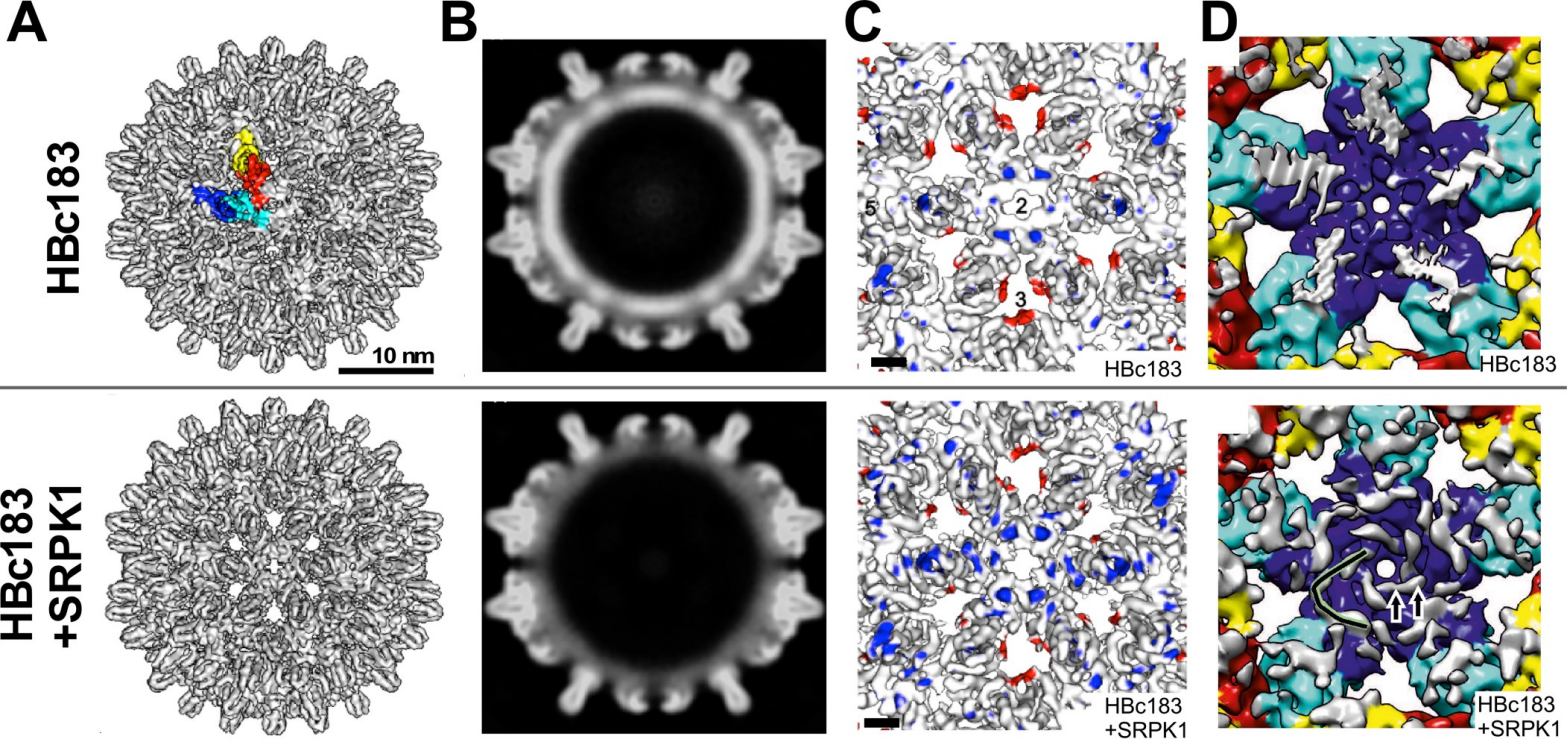
CryoEM comparison between non-phosphorylated and seven-fold CTD-phosphorylated HBc183 CLPs. **(A) Surface representations from image reconstructions at 7.8 Å and 7.9 Å resolution.** The A, B, C, and D monomers of one asymmetric unit in the T = 4 HBc183 CLP are colored blue, cyan, red and yellow. **(B) Density representations of equatorial slices.** Non-phosphorylated HBc183 CLPs display an unstructured shell of high density underneath the inner surface (top) which was absent from the SRPK1 coexpressed particles (bottom) with their much lower RNA content. **(C) Impact of seven-fold phosphorylation and/or low RNA content on the structure of the assembly domain.** The surface representations show close-ups of the two-fold symmetry axes at 7.8 Å and 7.9 Å resolution, sharpened with B-factors of -797 Å^2^ and -979 Å^2^, respectively. For clarity densities at radii <127 Å and small disconnected speckles were removed. Surface coloring reflects differences between the normalized unsharpened maps. Only differences with a confidence level of >99% (Student´s t-test) are shown. Colored areas highlight extra densities in non-phosphorylated (red) and phospho-HBc CLPs (blue). The length of the scale bar equals 2 nm. Numbers 2, 3 and 5 indicate the respectice symmetry axes. **(D) Ordered internal protein density in seven-fold phosphorylated HBc183 CLPs.** Shown are luminal close-ups of the five-fold symmetry axes; density accounted for by modelling the crystal structure of HBc149 CLPs (pdb: 1QGT) into the reconstructions is color-coded as in (A); not accounted for density is shown in white. Note the tube-like structures extending all the way to the feet of the spikes (one of the five highlighted as black continuous line) in the phospho-HBc CLPs. The extensions appear in direct contact with R112, part of the inner CLP lining (S11B Fig).

To directly assess the impact of seven-fold CTD phosphorylation and/or low RNA content on capsid structure we analyzed nonphosphorylated vs. SRPK1-phosphorylated HBc183 CLPs by cryoEM (S10A and S10 Fig) and calculated image reconstructions (Fig 8) at 7.8 Å (B = 707 Å^2^) and 7.9 Å (B = 979 Å^2^) resolution (S10C Fig). Most striking was a distinct shell of internal density exclusively in the non-phosphorylated particles (Fig 8B) which must represent CTDs plus packaged RNA. The relatively high B-factors suggested some structural heterogeneity within each CLP class, hence the gross features in the shells of unmodified vs. phosphorylated particles appeared very similar. However, in defined areas significantly different grey value distributions occurred (Fig 8C; see S2 Protocol for significance calculations). For the SRPK1 coexpressed CLPs they could be best described as reduced order in the N terminal regions which flank the spikes (Fig 8C bottom vs. top: less red color around the three-fold axes), plus an apparent stretching of the spike helices and the inter-dimer contact regions (Fig 8C bottom vs. top: more blue color within the dimer contours).

To improve resolution for fitting these differences into atomic models of HBc we vitrified additional aliquots of the same sample preparations which, fortuitously, had been stored for 1.5 years at 4°C. Corroborating the high stability of HBc CLPs, NAGE, SDS-PAGE and Phos-tag SDS-PAGE revealed no signs of degradation or loss of phosphorylation. Surprisingly, however, by 3D-classification the majority of these "aged" CLPs, especially the phosphorylated ones, grouped into a distinct class from the bulk of fresh particles (vitrified within two weeks post preparation) used above. The aged particle reconstructions had higher resolution and lower B-factors (6.3 Å and 6.6 Å, and 555 Å^2^ and 466 Å^2^, respectively, for non-phosphorylated vs. SRPK1-phosphorylated CLPs), in line with increased ordering with time. For the non-phosphorylated CLPs the differences were only minor but for the phospho-HBc particles they were pronounced. As in the comparison with fresh unmodified CLPs (Fig 8C) the freshly vitrified phospho-CLPs showed extended spikes and less ordered N termini (S11A Fig, top) whereas after ageing they appeared very similar to the non-phosphorylated ones (S11A Fig, bottom). This suggests a conformational maturation process, although not necessarily in the classic sense of gaining envelopment competence.

Fitting the HBc149-derived crystal structure 1QGT [16] into these reconstructions accounted for most density in the assembly domains but left unaccounted internal density close to the inner CLP surface, likely related to CTDs plus RNA in the non-phosphorylated CLPs yet only CTDs in the phospho-CLPs. The undisturbed view onto the inner shell surface of the latter particles revealed extensive tube-like structures around the 5-fold symmetry axes (Fig 8D, bottom vs. top). Connecting to the last visible residues in the crystal structure they probably represent residues from the linker plus the CTD. The respective density extended towards the feet of the spikes, with apparently direct contacts to R112 in both fresh and matured CLPs (S11B Fig). Their visibility implies rather stable interactions, perhaps including electrostatic binding between R112 and phosphorylated CTD residues in the phospho-CLPs (S9C and S9D Fig). Similar though less prominent extra density was also seen in the non-phosphorylated CLPs, although their high RNA content makes a clear distinction between nucleic acid and protein residues difficult (S11B Fig).

### The bulk of HBc183 in human hepatoma cells is similarly highly though non-identically phosphorylated as SRPK1-coexpressed HBc183

**Fig 9:**
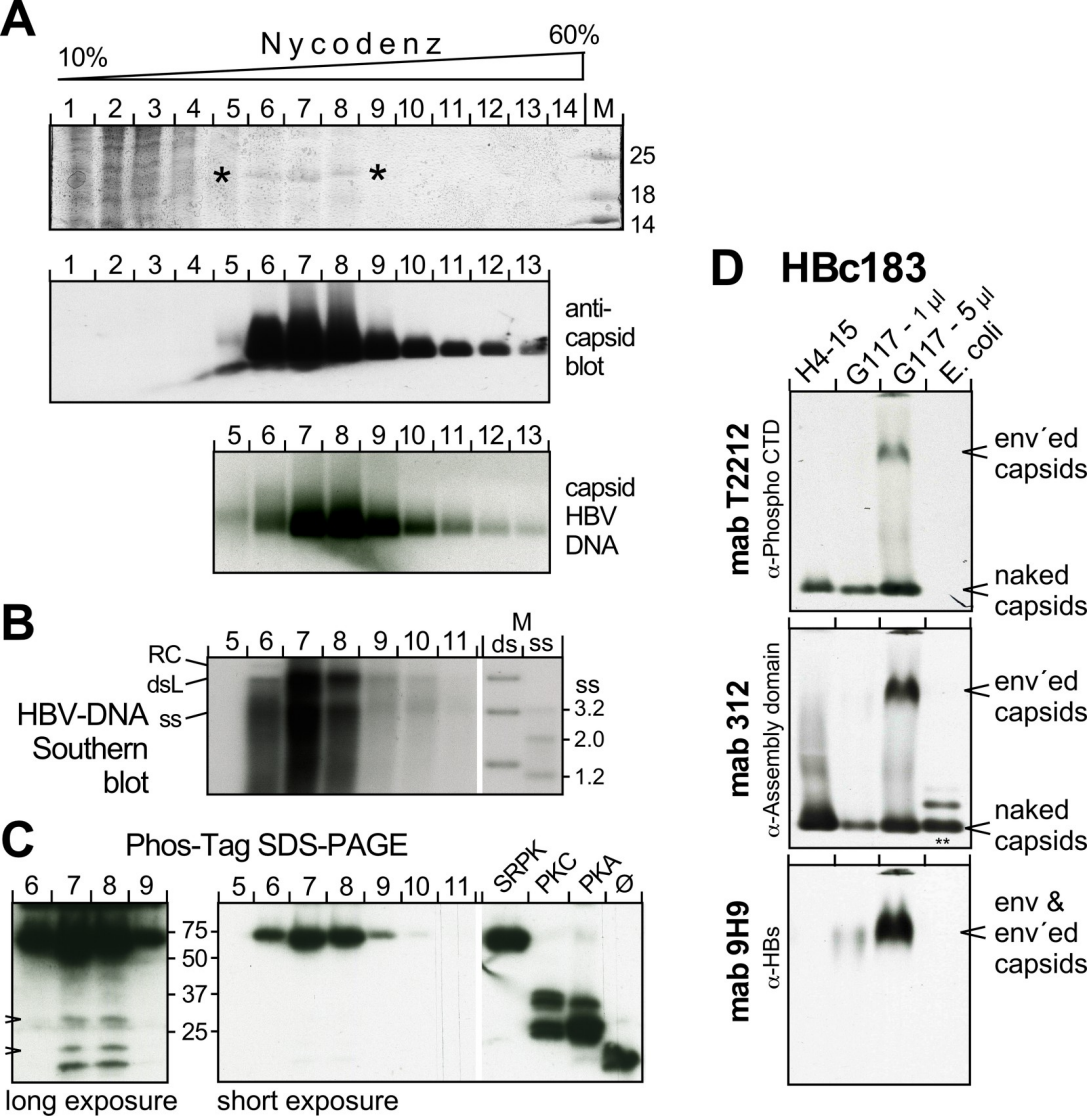
The bulk of HBc183 in capsids from human hepatoma cells is highly phosphorylated. **(A) Enrichment of particulate HBc by Nycodenz gradient sedimentation.** Cytoplasmic lysate from HBV producing HepG2.117 cells was sedimented through a Nycodenz gradient. Fractions were analyzed by SDS-PAGE and CB staining (top panel; asterisks mark a 21 kDa band possibly representing HBc183; the complete gel is shown in S12A Fig); by NAGE followed by immunoblotting with the anti-HBc assembly domain mAb 312 as PO conjugate (middle panel); and by hybridization with a ^32^P-labeled HBV probe. **(B) Southern blot for capsid-borne HBV DNA in individual gradient fractions.** M, DNA marker comprising HBV-specific fragments of the indicated sizes loaded in native ds form, or in heat-denatured ss form. **(C) Phos-Tag SDS-PAGE immunoblot.** Aliquots from the respective gradient fractions and recombinant HBc183 coexpressed with the indicated kinases, or not (ø), were separated by Phos-Tag SDS-PAGE and immuno-blotted with mAb 1D8. Short exposure showed one band with comparably strong retardation as SRPK1-phosphorylated HBc183. Longer exposure (left panel) revealed weak additional bands with mobilities similar to those of unmodified and PKC and PKA phosphorylated HBc183 (arrowheads). **(D) Enveloped capsids contain phosphorylated HBc183.** PEG-precipitated particles in supernatants from HepG2.117 cells, or from an HBc183 producing Huh7 line, H4-15 were analyzed by NAGE immunoblotting alongside *E*. *coli* HBc183 CLPs. The blot was sequentially probed with mAb T2212 (anti-phospho-CTD), mAb 312, and mAb 9H9 (anti-HBs). Note that T2212 detected only HBc from eukaryotic cells, including a low mobility species that comigrated with HBsAg and was absent from the H4-15 samples; it therefore represents enveloped capsids. Additional data employing mAb T2212 are presented in S12 Fig.

Recent data suggest that most HBc in mammalian cells from replicating as well as non-replicating vectors is phosphorylated [13, 52] yet the number of phospho-sites per HBc monomer is unknown. We therefore analyzed HBc in intracellular capsids from the stable HBV producing TetOFF HepG2.117 line [84] by Phos-tag SDS-PAGE, using our recombinant phospho-HBc183 proteins as markers. To minimize band distortions seen with crude cytoplasmic lysate we first enriched capsids by sedimentation through 10–60% (w/v) Nycodenz gradients (Fig 9A; S12A Fig). The presence of HBV-typical replicative DNA intermediates was corroborated by Southern blotting (Fig 9B); however, less vs. more mature species were barely separated and the presence of empty particles [54] remains to be addressed.

Regardless of this, Phos-tag SDS-PAGE showed a comparably strong retardation of the bulk HBc signals as for SRPK1-phosphorylated recombinant HBc183 (Fig 9C, short exposure). Longer exposure revealed several very weak intermediate mobility bands in the HepG2.117 samples, similar to those seen for CLPs coexpressed with PKA and PKC, and for non-phosphorylated CLPs; however, >95% of the band intensity was concentrated in the slowest migrating band. Though this would be in line with an SRPK1-like phosphorylation further investigation using the phospho-CTD specific mAb T2212 [51] indicated a non-identical phosphorylation pattern. As reported mAb T2212 did not recognize unmodified recombinant HBc183, yet it reacted strongly with the PKA- and some of the PKC-phosphorylated HBc species. Interestingly, fully SRPK1-phosphorylated HBc gave almost no signal whereas single alanine replacements of the phospho acceptor sites S162, S168 and, weakly, S176 and S178 but not S170 restored reactivity (S12 Fig). Hence the T2212 epitope appears to depend on the presence of phosphoryl groups at some positions, including S170, yet their absence from nearby sites.

To assess whether mAb T2212-reactive phospho-HBc species are present in enveloped particles we separated extracellular particles from HepG2.117 cells and from a new HBc183-only expressing Huh7 line by NAGE; recombinant HBc183 CLPs served as control (Fig 9D). Immunoblotting revealed a fast migrating band in the human cell-derived samples which corresponded to naked capsids, as indicated by comigration with bacterial HBc183 CLPs; as before, the latter did not react with mAb T2212 but were detected by the anti-assembly domain mAb 312 (marked by **). Importantly, mAb T2212 revealed a second, slower migrating band exclusively in the HepG2.117 samples which also stained with the anti-HBs mAb 9H9 [85]. Hence capsids containing phosphorylated HBc can be enveloped. More detailed conclusions will require separation from the naked capsids and efficient fractionation of enveloped capsids with differing nucleic acid content.

## Discussion

Dynamic (de)phosphorylation events are long thought of as enabling hepadnaviral core proteins to execute their multiple functions in the viral life-cycle but many details remained obscure. The tools developed in this study, especially in combination, are beginning to shed new light on these unresolved issues.

Our optimized *E*.*coli* coexpression system provides robust access to defined HBc species phosphorylated by individual kinases, as shown here for SRPK1, PKA and PKC. Varying the HBc part allows to pinpoint how specific mutations and/or the assembly status affect HBc´s substrate quality for a given kinase; in addition, the recombinant phospho-HBc proteins will make well-defined substrates to study phosphatases. Phos-tag SDS-PAGE was able to separate HBc species containing from no to seven phosphoryl groups which together with MALDI-TOF MS enabled us to map the seven SRPK1 target sites in the CTD. This, in turn, allowed correlating the number of CTD phosphoryl groups with CLP RNA content, stability and CTD exposure. Importantly, the new tools indicated that the bulk of HBc in human hepatoma cells is similarly highly phosphorylated as the recombinant SRPK1 phosphorylated protein, albeit in a non-identical pattern. Here we first discuss technical aspects of our study and then its implications for HBc phosphorylation in the viral life-cycle.

### Relevance of recombinantly phosphorylated HBc

Our MS data confirmed the absence from *E*. *coli* BL21 cells of enzymes capable of phosphorylating any residue in HBc183 (Figs 3 and 4; S5 and S6 Figs). Hence all previously reported properties of *E*. *coli* derived HBc CLPs relate to the fully unphosphorylated state. *Vice versa*, co-expressing a eukaryotic kinase then allows to single out how that specific kinase acts on HBc, as shown here for SRPK1 and per proof-of-principle for PKA and PKC.

There are still caveats for interpretation, including the concentration and ratio of kinase to HBc substrate in the bacteria, plus the potential sequestration of CTD-embedded target sites in the capsid interior. ATP is present in *E*. *coli* at concentrations of ≥1 mM [86] and thus not limiting. However, a low translation rate of a kinase compared to HBc, or poor solubility and/or low affinity for HBc could all affect phosphorylation efficiency. The higher levels of soluble SRPK1ΔNS1 (S3 and S4 Figs) likely explain the homogeneous seven-fold phosphorylation found here compared to the mixed phospho-HBc species seen upon coexpressing full-length SRPK1 [11]. An impact of target sequestration on phosphorylation efficiency is supported by the MS data for PKA coexpression which indicated predominantly three-fold phosphorylation of self-assembling HBc183 (S7 Fig), yet four- and five-fold phosphorylation of the non-assembling GFP-CTD protein (S6 Fig).

For SRPK1 the seven identified phosphorylation sites likely represent the maximum number; notably they include T160 (Fig 5) although SRPK1 is mainly considered as a serine kinase [72, 87]. Furthermore, the apparent stability of bacterial phospho-HBc in the absence of Phos-Stop inhibitor suggests the absence of phosphatases able to revert the mammalian-type phosphorylations.

A compromised substrate specificity in the *E*.*coli* system is unlikely because none of the eleven S and twelve T residues upstream of the CTD showed any indication of phosphorylation when HBc140 or HBc149 were coexpressed with SRPK1 (S7 Fig).

In sum, these data advocate a broad applicability of our recombinant phospho-HBc system to reveal the impact of individual kinases (and with adaptations, of phosphatases) on HBc.

### Feasible distinction of different phospho-HBc species by Phos-tag SDS-PAGE

Different from conventional SDS-PAGE Phos-tag SDS-PAGE resolved HBc species containing from none to five or six phosphoryl groups. While higher phosphorylation generally correlated with stronger retardation, discrimination amongst highly phosphorylated species was less clear, also owing to sequence context specific effects. (Fig 5A and 5B). It will be interesting to see whether variants with unusual Phos-tag mobility shifts also display specific biological features. For instance, two of the three single-site mutations causing lower than average retardation, S162A and S170A (Fig 5C), had the strongest negative impact on pgRNA encapsidation [47, 48, 88]. Coexpression with SRPK1 of additional HBc183 S/T>A mutants would provide a unique, comprehensive panel of reference proteins for more systematic investigations.

### Impact of sevenfold CTD phosphorylation on basic HBc particle properties: RNA content, detergent stability and CTD exposure

Uniform seven-fold phosphorylation by SRPK1 enabled to correlate a defined phospho-status with basic properties of HBc. Most striking was the drastic reduction in CLP RNA content (Figs 2, 5, 6 and 7). This has not been seen with partly phosphorylated HBc or with phosphorylation mimicking S/T>D,E variants. For instance, a variant termed 7E with seven S/T>E replacements in the CTD, expressed in HEK293 cells, reportedly contained still half as much RNA as nonphosphorylated bacterial CLPs [89]; similar results were seen for an all-S>D variant (S7D) expressed in *E*.*coli* [13]. Likely, the much stronger impairment of RNA binding by seven phosphoryl groups versus seven carboxyl groups is owed to their different chemistries. Seryl- and threonyl-phosphomonoesters are dibasic acids. With pKa values of 1.2 and 6.5 [90] even the second phosphoryl hydroxy group will be mostly deprotonated at physiological pH; hence each phosphoryl group can contribute nearly two negative charges. Seven-fold CTD phosphorylation would thus suffice to largely neutralize the 15 net positive charges (Fig 1), minimize the CTD´s general RNA binding capacity, and explain the low RNA content of the resulting CLPs.This interpretation is supported by inverse experiments in eukaryotic settings where complete inhibition of CTD phosphorylation by S/T>A mutations led to the formation of non-specifically RNA-filled rather than empty HBc CLPs [13, 89]. Practically, low RNA CLPs from SRPK1 coexpression in *E*. *coli* of full-length HBc variants bearing heterologous sequences could be of interest for vaccine applications [74].

Seven-fold phosphorylation and low RNA content had little impact on CLP stability against SDS (Fig 6) whereas trypsin treatment revealed specific phosphorylation and/or RNA content-dependent differences; their independence from the F97L mutation suggests that the structural changes causing the premature virion secretion phenotype of the F97L variant are either very subtle or not sufficiently long-lived to be observed [91], as corroborated in a recent high resolution cryoEM study [92].

Non-phosphorylated CLPs yielded one major HBc149-like product ("band 4" in Fig 7B and 7C), whereas the SRPK1-phosphorylated CLPs gave two additional, stable intermediates (Fig 7C and 7D; S9 Fig). These data are reminiscent of but also distinct from artificially RNA-depleted HBc-EEE vs. WT CLPs [37]; there, a band similar in size to our band 3 also accumulated over time, however, in both wild-type and HBc-EEE CLPst. The more pronounced differences in our experiments may relate to the higher number of modifications per CTD plus the chemical differences outlined above. Two models that could explain formation of distinct trypsin processing products (S9C and S9D Fig) would invoke either complete extrusion of some CTDs, or the looping-out of selective trypsin target sites with the very C terminal CTD residues still internally disposed. In the phospho-HBc CLPs this could be mediated by electrostatic interactions between the phosphoryl groups and positively charged side-chains in the CTDs and/or on the inner CLP lining, as proposed for HBc-EEE CLPs [37]. Maintained integrity and RNA content of the trypsin-treated CLPs (Fig 7B, 7C and 7E) appear in better accord with the looping-out model.

The current cryoEM reconstructions did not resolve this issue but they revealed distinct phosphorylation-dependent differences. The lack in SRPK1-coexpressed HBc183 CLPs of internal RNA density (Fig 8B) enabled allocating the remaining extra density to the linker plus CTD residues which formed tube-like structures around the five-folds, in apparent contact with residues lining the capsid lumen, i.e. R112 (S11B Fig). Freshly vitrified phospho-CLPs showed well detectable local differences in their assembly domains compared to unmodified CLPs which largely disappeared upon long-term storage. In particular the very N termini adopted a more ordered conformation, as in both fresh and aged unmodified CLPs. A potential physiological relevance of this slow rearrangement in the phospho-CLPs remains to be determined. Notably, though, replacement of the N-proximal P5 lowered virus secretion [93] and P5 is close in space to residues 95–97 implied in interactions with L protein [94].

### Implications of high-level HBc CTD phosphorylation for the HBV life-cycle

The bulk of HBc183 in capsids from human cells was strongly retarded in Phos-tag SDS-PAGE (Fig 9C, S12B Fig), in line with an independent report [13]. Comparison with the recombinant phospho-HBc proteins indicated an SRPK1-like seven-fold phosphorylation status, in line with a physiological role of SRPK1 as a HBc kinase [34, 43, 71]. However, Phos-tag retardation alone would be also compatible with a slightly lower or higher phosphorylation extent (Fig 5). Also, the strong reactivity of mAb T2212 with the human cell-derived but not the recombinant phospho-HBc (S12 Fig) indicates at least one phosphorylation-dependent difference. If SRPK1 was indeed the main HBc kinase, (a) compatible phosphorylation pattern(s) could arise from one or few selective dephosphorylation events. Alternatively, however, the combined action of other kinases (and phosphatases) could lead to a similar pattern.

Notably, mAb T2212 detected phosphorylated HBc in enveloped particles (Fig 9), as proposed for empty virions [52]. Further exploration using our new tools, with more efficient separation of the various HBV particles [54], will help to accurately ascribe specific phosphorylation patterns to individual particle types.

**Fig 10:**
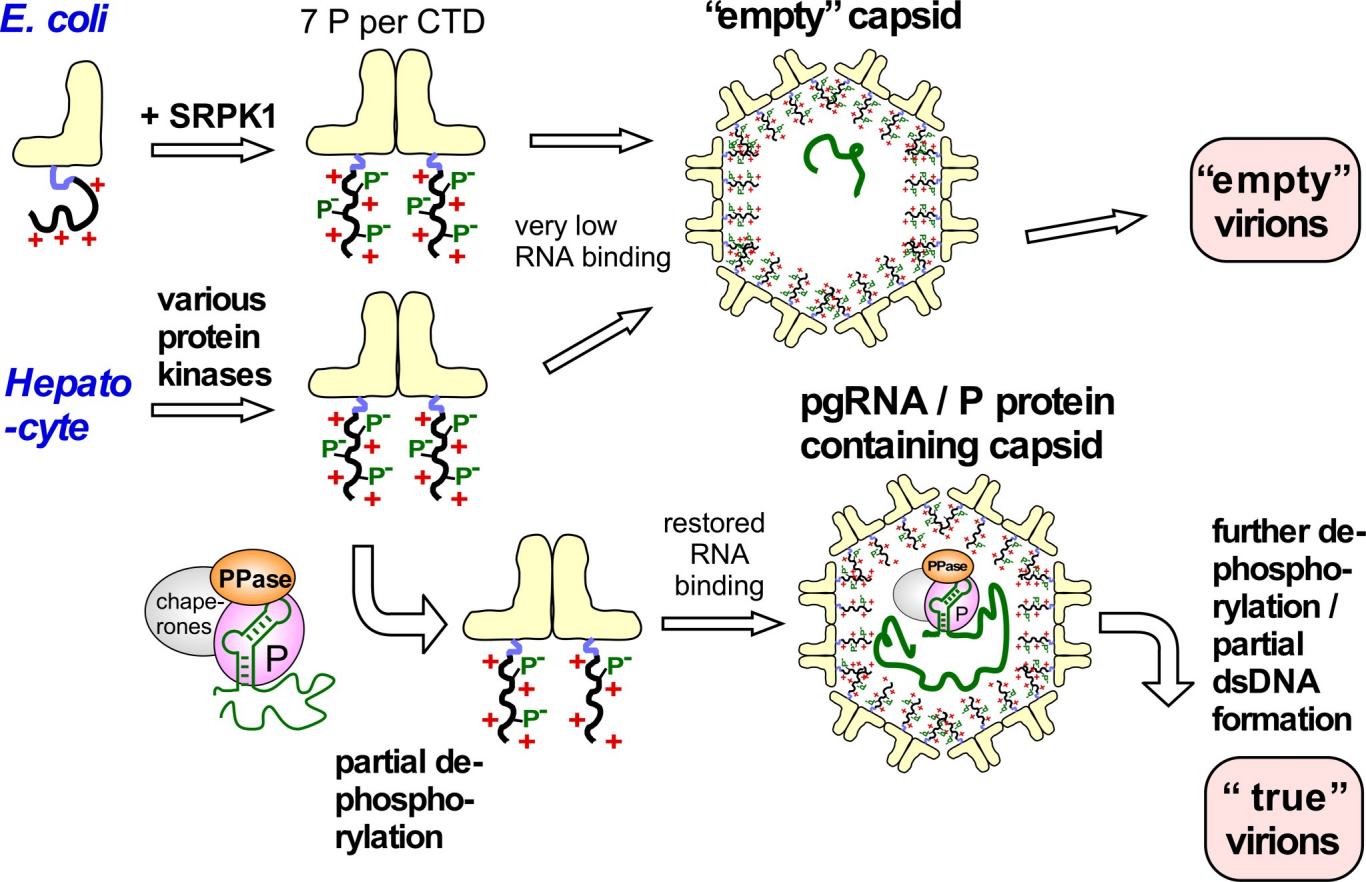
Implications of the high HBc phosphorylation—Low CLP RNA content correlation for specific pgRNA encapsidation in HBV infection. In *E*.*coli* nonphosphorylated HBc183 CTDs have maximal positive charge and thus maximal electrostatic RNA binding capacity, generating RNA-filled CLPs (Fig 1B). Seven-fold phosphorylation by SRPK1 neutralizes most positive charges, leading to virtually empty capsids. The similarly strong Phos-tag retardation of most HBc183 from human cells indicates similarly high phosphorylation, whether by SRPK1 or a combination of kinases. Strongly reduced RNA binding favors formation of empty capsids and then empty virions, perhaps the dominant pathway in vivo [52]. The backside of avoiding irrelevant RNA packaging is a loss in specific pgRNA interaction capability. As blocking just one of the seven SRPK1 phosphorylation sites restored substantial RNA packaging in bacteria we propose that the pgRNA/P protein complex, besides other host factors, also carries a protein phosphatase (PPase) activity. This PPase would dephosphorylate only nearby HBc CTDs, and thus locally unleash their RNA binding potential in proximity to pgRNA. This would go on with further HBc dimers until the shell is completed. Progressive dephosphorylation of the now internal CTDs by the PPase activity could maintain electrostatic homeostasis, especially during the near doubling of negative charges associated with dsDNA formation. Targeted nucleocapsid destabilization upon infection of a new cell could occur by CTD re-phosphorylation and completion of plus-strand DNA.

At any rate does the high phosphorylation level of most HBc183 in human cells imply a similar suppression of general RNA binding capacity as in recombinant SRPK1 phosphorylated HBc183. As outlined in Fig 10, in favor of viral replication this would counteract encapsidation of irrelevant RNAs [13, 89] yet also impair pgRNA packaging, giving empty capsids and empty virions. This dilemma could plausibly be resolved by partial dephosphorylation, possibly at just one site (Fig 5C). Most effective would be a phosphatase activity associated with the pgRNA—P protein complex, providing spatio-temporal control of the dephosphorylation event(s); HBc dephosphorylation coincident with pgRNA packaging has indeed very recently been suggested [95]. HBc dimers joining the nascent capsid, but not bulk HBc, could then as well become partly dephosphorylated, enabling stable packaging of the entire 3.5 kb pgRNA. With progressing dsDNA synthesis the co-packaged phosphatase activity could gradually release more CTD phosphoryl groups until this electrostatic buffer is emptied [13, 26, 75]. One might further speculate that timely envelopment blocks the supply of dNTPs into the capsid before the (+)-DNA gap is completely filled, and that this represents the most stable state for a DNA containing nucleocapsid ready to leave the cell as virion. Conversely, after infection of a new cell continued DNA synthesis plus re-phosphorylation might create an excess of negative charge and destabilize the capsid in preparation for uncoating.

More work will be needed to substantiate this model, but the concept as such lets previous statements on the importance of HBc phosphorylation for HBV replication appear oversimplified. Rather than directly promoting pgRNA encapsidation HBc phosphorylation seems to act indirectly by blocking competing interactions with non-specific RNA. In turn, with high-level HBc phosphorylation as default, dephoshorylation becomes as important for viral replication as phosphorylation. Hence production of replication-competent HBV nucleocapsids appears to depend on an intricately balanced level of HBc phosphorylation. The excess of empty over genome-containing HB virions [52, 60] indicates that proper execution of this program is delicate; hence even small perturbations might have severe consequences, inviting therapeutic exploitation, e.g. by kinase [96] or phosphatase inhibitors. However, such strategies will require a deeper understanding of the involved host enzymes and the substrate properties of HBc for which our study provides valuable new tools. On the basic side, these tools can feasibly be adapted to other viruses, including but not limited to relatives of HBV [97]. Preliminary data indicate, for instance, that the core protein of DHBV, a virus capable of replicating in human cells [98], is also highly phosphorylated (at eight yet to be mapped sites) by human SRPK1 in the recombinant coexpression system whereas the core protein of the fish Cichlid nackedna virus (CNDV; [97]) is not. Compatibility with the cellular kinases and phosphatases may thus well be a determinant of viral host and tissue tropism.

## Materials and methods

### Plasmid constructs

HBc expression vector pET28a2-HBc183 [69] carries a T7 promoter controlled synthetic HBc gene [68] for a genotype D (GenBank: V01460.1) core protein. The HBc183opt ORF used here encodes the same aa sequence but was adapted to *E*. *coli* codon usage (GeneOptimizer software; ThermoFisher/GeneArt), increasing CLP yields ~3-fold (S1 Fig). The new pRSF expression vectors are based on pRSFDuet-1 (Novagen) which harbors two T7 promoter cassettes and the *lacI* repressor gene for IPTG inducible coexpression of two ORFs. For HBc mono-expression the HBc183opt ORF and its derivatives were usually inserted after the 5´ T7 promoter, with simultaneous deletion of the 3´ T7 promoter cassette (plasmids pRSF_T7-HBcNNNopt; NNN specifies the last HBc aa present). For coexpression, the upstream T7 promoter was replaced by a Tet promoter, and in addition a gene for the Tet repressor was inserted into the plasmid (S2A Fig) for separate inducibility by anhydrotetracycline (AHT). Kinase ORFs were inserted under Tet promoter control, HBc ORFs under T7 promoter control (plasmids pRSF_Tet-X_T7-HBcNNNopt, where X denotes the ORF under Tet promoter control). Employed kinase ORFs encoded an N-terminally His6-tagged version of SRPK1ΔNS1 [72]; the catalytic subunit alpha isoform 1 (aa 1–351) of PKA (NP_032880.1); and the C-terminal catalytic domain (aa 397–740) of human PKC theta isoform X1 (XP_005252553.1). The latter two ORFs had previously been expressed in bacteria [79, 80] and here were obtained as *E*. *coli* expression optimized DNA strings (ThermoFisher/GeneArt). In the non-assembling CTD control constructs the HBc aa 1–145 part was replaced by the ORF for N-terminally His6-tagged eGFP [99] followed by a Gly_2_ linker and a recognition site for TEV protease (see Fig 4A). Cloning was done by conventional restriction-based methods or using the Q5 mutagenesis kit (NEB). All constructs were verified by Sanger sequencing.

### Expression of recombinant proteins in *E*. *coli* and HBc CLP enrichment

Expression of HBc CLPs followed previously described procedures [99, 100], as detailed in reference [101]. In brief, *E*. *coli* BL21*CP served as expression host. T7 promoter and Tet promoter driven expression from the pRSF plasmids were induced by IPTG (1 mM final concentration) and/or 0.2 μg/ml AHT, respectively; cultures (usually 200 ml) were then shaken for 12–16 h at 20–25°C. Cell lysis included treatment with lysozyme, Triton X-100 and benzonase (Merck-Millipore) in the presence of protease-inhibitor cocktail (Roche) with subsequent sonication; in kinase coexpressions, Phos-Stop phosphatase inhibitor (Roche) was included. Cleared cell lysates were subjected to sedimentation (TST41.14 rotor; 41,000 rpm for 2 h at 20°C) through 10%-60% sucrose step gradients in TN150 buffer (25 mM Tris/HCl, 150 mM NaCl, pH 7.4). For longer term storage at -80°C, peak fractions were dialysed into storage buffer (50 mM Tris/HCl pH 7.5, 5 mM EDTA, 5% (w/v) sucrose, 2 mM DTT). Isolated CTD peptides from TEV-cleavable GFP fusions were obtained as detailed in S1 Protocol.

### Native agarose gel electrophoresis (NAGE)

NAGE was performed in 1% agarose gels in 1x TAE buffer (40 mM Tris, 20 mM acetic acid, 1 mM EDTA) as previously described [9, 23, 99]. For routine detection of nucleic acids gels contained 0.5 μg/ml ethidium bromide (EB); subsequent protein staining was done using Coomassie Brilliant Blue R250 (CB) followed by extensive destaining in fixing buffer (50% MeOH, 7% acetic acid, v/v in H_2_O).

Alternatively, gels run without EB were stained for RNA using 1x Sybr Green 2 (SG2) RNA stain in TAE buffer (from 10,000x in DMSO; FMC Bioproducts), followed by protein staining with ready-to-use Sypro Ruby (SR) Protein Gel Stain (BioRad). Fluorescence signals were recorded using a laser Scanner (Typhoon FLA 7000; GE Healthcare) set at excitation 473 nm / filter Y520 nm (SG2) and 473 nm / filter O580 nm (SR). Signals were quantified using ImageQuant software. Higher quantitation accuracy was achieved by several adapations of the protocol, as described next.

### HBc CLP RNA content by fluorescence intensity of RNA versus protein staining after NAGE—Improved protocol

Critical issues for robust signal quantitation included weak SG2 staining (especially for low RNA content CLPs), concentration-dependent variations in SR signal intensities, SG2 to SR signal carry-over during Laser scanning, and high background staining. Implemented countermeasures were to use gels no thicker than 5–6 mm; loading similar protein amounts of different CLPs and possibly two different amounts of the same CLPs; and including on each gel a well-characterized wild-type CLP preparation as standard to account for variations in staining intensity. SG2 staining over background was improved by doubling the dye concentration (1:5,000 dilution), a 1 h staining period, plus ≥3 washes with TAE buffer prior to SG2 laser scanning. For improved SR staining gels were washed twice in fixing buffer, after which the SG2 staining was no longer detectable; fixation also prevented band broadening by diffusion during the subsequent overnight incubation in SR protein gel stain. After two additional washes in distilled water SR fluorescence was recorded as described above. For each gel, the ratio of SG2: SR fluorescence intensity in the wild-type HBc183 standard was set to 100% to which the respective intensity ratios from the test CLPs were normalized. Applying this procedure reduced the calculated relative RNA content of CTD-less and SRPK1-phosphorylated CLPs from >25% of that of unmodified HBc183 CLPs (Fig 2C) to ≤10% (Fig 5C, S8 Fig).

### HBc CLP RNA content by UV/VIS spectrometry

RNA content was calculated from CLP sample absorbances at 260, 280, 340 and 360 nm [73]. For high purity CLP preparations, e.g. HBc183 CLPs from two sequential sucrose gradients [101], ten-fold dilutions in H_2_O were measured in 1 cm path length cuvettes in an Ultrospec 7000 instrument (GE Healthcare). For single gradient CLP preparations which may contain free RNA [101] aliquots from the respective fractions (routinely containing 0.5–4 mg/ml of HBc protein) were incubated with 1/100 (w/w) of RNase A for 30 min at room temperature, then dialyzed (30 kDa MWCO) against TN 150 buffer. UV/VIS absorbances of the resulting solutions were then monitored without further dilution in a QIAxpert instrument operating with 0.1 cm path length (2 μl volume) microcuvettes. Molar nucleotide per core protein monomer (N/P) ratios were calculated as described [73].

### Phos-tag SDS-PAGE

Mn^2+^-Phos-tag SDS-PAGE was performed by adding Phos-tag acrylamide AAL-107 (Wako Pure Chemical Corporation) plus MnCl_2_ to the acrylamide solutions for conventional Lämmli SDS-PAGE resolving gels, as recommended by the manufacturer. In our hands, the best separation of differently phosphorylated HBc183 species was obtained using 15% acrylamide gels supplemented with 75 μM Phos-tag acrylamide and 150 μM MnCl_2_.

### Immunoblotting

Immunoblotting after NAGE or conventional SDS-PAGE was performed as previously described [69, 102]. Low transfer efficiency to polyvinylidene difluoride (PVDF) membrane after Phos-tag SDS-PAGE gels was improved by sequentially soaking the gels for 10 min each in transfer buffer A (39 mM glycine, 48 mM Tris, 3.75% (w/v) SDS, 20% (v/v) MeOH) containing 10 mM EDTA, 1 mM EDTA, and no EDTA, plus the use of a wet blot rather than a semi-dry transfer system. HBc specific antibodies employed were the anti-assembly domain mouse mAbs 312 [103] and 1D8 [82], both recognizing a linear epitope exposed on intact CLPs and SDS-denatured HBc protein; the capsid-specific mAbs 275 [104] and 3120 [105], obtained from Tokyo Future Style, Inc. (catalogue no.: 2AHC22); and the anti-phospho-CTD mAb T2212 [51], also from Tokyo Future Style (catalogue no.: 2AHC23). HBsAg on NAGE blots was detected by human mAb 9H9 [85] which recognizes a conformational epitope in S. Bound mAbs were visualized using horse raddish peroxidase (PO), either as direct mAb conjugate or via appropriate secondary antibody-PO conjugates, and chemiluminescent substrates.

### HBc CLP sensitivity against SDS

About 5 μg of sucrose gradient enriched HBc CLPs were incubated for 30 min with increasing amounts of a 6x DNA loading buffer containing 0.48% SDS (NEB Purple) to yield final SDS concentrations from 0.024% to 0.24%; samples incubated in a non-denaturing 6x loading buffer served as control. Reaction products were monitored by NAGE in 1% gels; EB fluorescence signals were recorded using a laser scanner (excitation 532 nm / filter O580 nm). Proteins were subsequently stained by CB.

### HBc CLP sensitivity against trypsin

For partial trypsin digests the respective HBc CLPs were incubated with 1/100 (w/w) the amount of sequencing grade trypsin (Promega) in TN50 buffer (25 mM Tris/HCl, 50 mM NaCl, pH 7.5) at 30°C. At various time points aliquots were withdrawn and digestion was stopped by adding 4-(2-aminoethyl)-benzenesulfonyl fluoride hydrochloride (AEBSF; Applichem) to a final concentration of 0.2 mM. Formation of cleavage products and intactness of particles were analyzed by SDS-PAGE and NAGE.

### Mass spectrometry (MS)

MS analyses were performed at the Institut de Biologie et Chimie des Protéines, UMR5086, CNRS/Université Lyon 1, France on a Sciex Voyager DE-PRO MALDI-TOF instrument, using sucrose gradient enriched CLPs diluted 1:100 (v/v) in sinapic acid as matrix.

### Electron microscopy and image processing

Electron microscopy was carried out in the Edinburgh, UK, cryoEM facility. Detailed procedures for sample preparation, micrograph recording, particle selection, image processing, refinement, difference map calculation, and molecular fitting are given in S2 Protocol. In brief, gradient-enriched CLP samples were vitrifed as described [106] using a manual freezing apparatus with an environmental chamber [107] at room temperature. Before use, grids (Quantifoil R1.3/1.2) were glow discharged in air for 1 min with a current of 30 μA using a Quorumtec sputter coater. Micrographs were recorded on a FEI Tecnai F20 microscope operated at 200 kV and a 8192 pixels x 8192 pixels CMOS camera (TVIPS F816). Only particles with round shape and crisp appearance were subsequently used for reconstructions. Resolution and B-factors were calculated with Relion [108]. Fitting was done using Chimera [109], based on pdb 1QGT [16].

### Human cells and culture conditions

The human hepatoma cell line HepG2 and its TetOFF HBV derivative HepG2.117 [84] were cultured as described [84, 110]. The stable, constitutively HBc183 producing cell line H4-15 was derived from the human hepatoma cell line Huh7 by CRISPR/Cas9-mediated homologous recombination of a HBc expression cassette into the AAVS1 locus (Peter Zimmermann, MSc thesis; University of Freiburg, 2017).

### Enrichment of HBV particles from human hepatoma cells

Cytoplasmic HBV capsids were obtained from TetOFF HepG2.117 cells cultured for 10 days in the absence of doxycycline [84]. Cytoplasmic lysates were prepared using NP40 lysis buffer (50 mM Tris/HCl, 140 mM NaCl, pH 8, with 0.5% (v/v) NP40 detergent) supplemented with Phos-Stop. To enrich capsids, lysates were sedimented through a 10% - 60% (w/v) Nycodenz step gradient for 2 h at 4°C in a TST 41.14 rotor at 41,000 rpm (302,500 g) and harvested in 14 fractions. Detection of HBV DNA in capsids and by Southern blotting was performed using ^32^P labeled HBV DNA probes as described [84, 111]. Extracellular particles in the culture supernatants of HepG2.117 and H4-15 cells were enriched by precipitation with PEG8000 [84, 112].

### Statistical analyses

Unless indicated otherwise data are expressed as mean ± standard deviation (SD) from ≥3 experiments. Comparisons between multiple groups were performed using One-way ANOVA and Tukey´s post test (Graphpad Prism 5). Differences between means of two paired groups were evaluated using Student's *t*-test. P-values of p<0.05 were regarded as statistically significant.

## Supporting information

S1 Protocol Isolation of HBc CTD peptides from GFP-CTD fusion proteins coexpressed in *E. coli* with mammalian protein kinases. (PDF)

S2 Protocol Electron microscopy and image reconstruction. (PDF)

S1 Fig Increased yield of HBc183 CLPs in *E. coli* by codon usage adaptation. (PDF)

S2 Fig Vector system for regulated coexpression of two gene products in *E. coli*. (PDF)

S3 Fig Efficient coexpression in *E.coli* of HBc183_F97L CLPs with SRPK1. (PDF)

S4 Fig Separation of HBc183 CLP-associated His6-SRPK1ΔNS1 under semidenaturing conditions. (PDF)

S5 Fig MS characterization of HBc183 expressed in the absence vs. presence of SRPK1ΔNS1. (PDF)

S6 Fig MS analysis of CTD peptides confirms seven SRPK1 phosphorylation sites in the HBc CTD and distinctly fewer sites for PKA. (PDF)

S7 Fig Predominant number of phosphoryl groups in kinase coexpressed wild-type HBc183 and CTD derivatives by MS analysis. (PDF)

S8 Fig Differential impact of kinase coexpression and phosphorylation site mutations on CLP RNA content. (PDF)

S9 Fig Schematic models for differential trypsin sensitivity of CTDs in unmodified vs. seven-fold phosphorylated HBc183 CLPs. (PDF)

S10 Fig CryoEM analysis of non-phosphorylated vs. highly phosphorylated HBc183 CLPs. (PDF)

S11 Fig Long-term storage induces a more ordered structure in SRPK1- phosphorylated CLPs. (PDF)

S12 Fig Similarly high though non-identical phosphorylation of HBc183 in human cells vs. SRPK1-phosphorylated HBc183. (PDF)
