# Supplementary material for: Hepatitis B virus core protein phosphorylation: Identification of the SRPK1 target sites and impact of their occupancy on RNA binding and capsid structure
Source: PLoS Pathog. 2018 Dec 19;14(12):e1007488. doi: 10.1371/journal.ppat.1007488 (PMC6317823; doi:10.1371/journal.ppat.1007488)
Supplement: S1 Protocol — (PDF) [file ppat.1007488.s001.pdf]

S1 Protocol. Isolation of HBc CTD peptides from GFP-CTD fusion proteins coexpressed in *E. coli* with mammalian protein kinases

The prime objective was to increase the accuracy of MS-based determination of the number of phosphoryl groups on kinase-coexpressed HBc by reducing the mass of the relevant fragment, i.e. the CTD. For specific cleavage of the intact CTD which is highly sensitive to trypsin-like proteases we fused the CTD sequence through a tobacco etch virus (TEV) protease site to N-terminally His6-tagged eGFP (1). For kinase co-expression we employed analogous pRSF vectors as for HBc plus kinase coexpression (S3 Fig), except that the terminal Cys on the CTD was mutated to Ala (C183A in HBc numbering) to minimize oxidation-related issues. In addition, the His-tag sequence on the kinases was deleted (Fig 4A) to prevent IMAC co-purification with the NHisGFP-CTD fusion protein; however, even then did separation of SRPK1 require semi-denaturing conditions (see S4 Fig and below).

Expression in *E.coli* BL21*Cp cells and cell lysis were performed as for HBc proteins, except that all steps were conducted on ice and in the presence of 0.2 mM AEBSF to block tryptic activities. The cleared lysates were adjusted to a final concentration of 6 M urea, then subjected to Ni^{2+}-IMAC using a Ni-NTA (Qiagen) column equilibrated in TN150 buffer (25 mM Tris/HCl, 150 mM NaCl, pH 7.4) containing 6 M urea and 0.2 mM AEBSF. Columns were developed using 6 M urea in TN150 containing 20 mM, 50 mM, 250 mM, 800 mM imidazol. As the eGFP chromophore remained stable, the green-colored fractions eluting at 250 mM imidazole were pooled, and the buffer was exchanged against TN400 (25 mM Tris/HCl, 400 mM NaCl, pH 7.4; 0.2 mM AEBSF) without urea by repeated concentration and dilution in Amicon centrifugal ultrafiltration devices with 10 kDa molecular weight cut-off (MWCO); lower salt conditions promoted formation of a precipitate in the subsequent TEV protease reaction. After concentration to ~0.5-1 mg/ml the fusion proteins were incubated overnight at 4°C with 1% (w/w) of home-made His-tagged TEV protease (2), again in the presence 0.2 mM AEBSF which did not inhibit TEV protease activity. When cleavage had sufficiently progressed the entire reaction was resubjected to Ni^{2+} IMAC in TN400 buffer. As desired, the protease-released His-tag-less CTD peptides were found in the flow-through whereas the other components, all carrying His-tags, were largely retained. CTD peptides in the flow-through were concentrated ~10-fold using Amicon devices with 3 kDa MWCO; a final ultrafiltration of the concentrate through a 30 kDa MWCO device removed most of the residual larger contaminants. SDS-PAGE analysis of the end products from coexpression with SRPK1ΔNS1, PKA, or no kinase vs. a chemically synthesized CTD peptide (sCTD) showed for all samples one major band comigrating with the 10 kDa marker (Fig 4C and S6A Fig). This was due to aberrant electrophoretic mobility, as confirmed by subsequent MALDI-TOF MS.

Supplementary references to S1 Protocol:

(1) Walker A, Skamel C, Nassal M. SplitCore: an exceptionally versatile viral nanoparticle for native whole protein display regardless of 3D structure. Sci Rep. 2011;1:5

(2) Walker A, Skamel C, Vorreiter J, Nassal M. Internal core protein cleavage leaves the hepatitis B virus capsid intact and enhances its capacity for surface display of heterologous whole chain proteins. J Biol Chem. 2008;283(48):33508-15
