# Supplementary material for: Hepatitis B virus core protein phosphorylation: Identification of the SRPK1 target sites and impact of their occupancy on RNA binding and capsid structure
Source: PLoS Pathog. 2018 Dec 19;14(12):e1007488. doi: 10.1371/journal.ppat.1007488 (PMC6317823; doi:10.1371/journal.ppat.1007488)
Supplement: S2 Protocol — (PDF) [file ppat.1007488.s002.pdf]

S2 Protocol. Electron microscopy and image reconstruction

Electron microscopy. Vitrified samples were transferred with a Gatan 626 cryo transfer holder into an FEI F20 electron microscope. The microscope was operated at 200 kV either with the C2-aperture at 50 µm and a spot size of 1 or with the C2-aperture at 100 µm with a spot-size of 3 to 5. Beam-tilt was corrected by coma-free alignment at the beginning of every session. Micrographs were recorded with a conventional CMOS camera (TVIPS F816, 8192 pixels x 8192 pixels) with a catalase calibrated pixel size of 1.53 Å. Exposure times were between 1-2 s and electron doses were between 13 and 20 e/Å^2. The microscope was operated semi-automatically using EM-tools (TVIPS GmbH). Eucentric height was adjusted whenever the accumulated displacement of the stage was larger than 50 µm. Image shifts for recording the final micrographs were smaller than 100 nm to minimize the beam tilt that is caused by the none-parallel illumination conditions of the F20.

Image processing. Particles were manually selected with e2-boxer (1). Typically, about 50% of all particles on a micrograph were selected for further processing. The defocus of each micrograph was determined with ctffind3 (2). Particle images were boxed from micrographs with Relion (3,4). The dimensions of the particle images were 300 x 300 pixels. All subsequent image processing was done with Relion. Particle images were normalized in their grey value distribution. Poorly aligning particle images were identified in 2D-classification and excluded from further processing. The remaining particles were processed with autorefine3D using a previously determined map of HBc183 CLPs as starting reference. Auto-refinement was followed by 3D-classification to identify major sub-groups in a population of particles or to test whether conformational differences were sufficiently large and consistent to be recognized *in silico*. Selected classes were further processed with auto-refine, refining the orientations of two halves of the data independently to avoid over-fitting (4). Resolution was determined with Relion from two independently processed halves of the data (gold standard refinement). For solvent flattening a smooth spherical shell was applied (r_{in}=90 Å; r_{out}=200 Å). Fourier shell correlation was corrected for the contribution of the mask (5). The resolution is given at the spatial frequency where the Fourier shell correlation drops below 0.143 (6).

B-factors were estimated, also with Relion, by correcting the reconstruction for the modulation transfer function (mtf) of the camera and then determining the B-factor as the linear slope of a Guinier plot for spatial frequencies larger than 1/10 A^{-1} after correction for the noise contribution (6). The mtf of our camera was determined as described (7) using the standard beam stop of the EM as sharp edge.

Student´s t-tests and difference maps. For assessing the significance of the differences between two reconstructions, we calculated Student´s t-tests. For each reconstruction we calculated 5 maps from a different subset of particles. The maps included spatial frequencies up to 1/8 Å^{-1}. The maps were used to calculate t-test values for each pixel of the map which were then used to identify areas differing by at least 0.25σ of the grey value distribution with a t-test value of p>99%. This information was used to generate a binary mask to identify areas with statistically significant differences (Fig 8C).

Differences were calculated between grey value normalized maps calculated to 8 Å resolution (Relion, no-B-factor sharpening). In Fig 8C difference maps were multiplied with the binary mask generated from the t-test map to identify the statistically significant differences. Maps of statistically significant differences were used to color the surface representations of HBc183 and phospho-HBc183 CLPs in Fig 8C. In Fig S11A surfaces are colored by the differences without significance testing. The color-

coding highlights differences of ≥2σ of the grey-value distribution of the maps; 2σ was also used as threshold for calculating the surface representation in S11A Fig.

Fitting. Two different structures of the asymmetric unit of HBc, pdb: 1QGT (8) and pdb: 3J2V (9), were manually placed at the appropriate position of the respective map using Chimera (10) followed by optimization with the "fit in map" option; 1QGT matched consistently better to our maps than 3J2V.

Supplementary references to S2 Protocol:

(1) Tang G, Peng L, Baldwin PR, Mann DS, Jiang W, Rees I, et al. EMAN2: an extensible image processing suite for electron microscopy. J Struct Biol. 2007;157(1):38-46

(2) Mindell JA, Grigorieff N. Accurate determination of local defocus and specimen tilt in electron microscopy. J Struct Biol. 2003;142(3):334-47

(3) Scheres SH. RELION: implementation of a Bayesian approach to cryo-EM structure determination. J Struct Biol. 2012;180(3):519-30

(4) Scheres SH, Chen S. Prevention of overfitting in cryo-EM structure determination. Nat Methods. 2012;9(9):853-4

(5) Chen S, McMullan G, Faruqi AR, Murshudov GN, Short JM, Scheres SH, et al. High-resolution noise substitution to measure overfitting and validate resolution in 3D structure determination by single particle electron cryomicroscopy. Ultramicroscopy. 2013;135:24-35

(6) Rosenthal PB, Henderson R. Optimal determination of particle orientation, absolute hand, and contrast loss in single-particle electron cryomicroscopy. J Mol Biol. 2003;333(4):721-45

(7) Ruskin RS, Yu Z, Grigorieff N. Quantitative characterization of electron detectors for transmission electron microscopy. J Struct Biol. 2013;184(3):385-93

(8) Wynne SA, Crowther RA, Leslie AG. The crystal structure of the human hepatitis B virus capsid. Mol Cell. 1999;3(6):771-80

(9) Yu X, Jin L, Jih J, Shih C, Zhou ZH. 3.5A cryoEM structure of hepatitis B virus core assembled from full-length core protein. PLoS One. 2013;8(9):e69729

(10) Pettersen EF, Goddard TD, Huang CC, Couch GS, Greenblatt DM, Meng EC, et al. UCSF Chimera - a visualization system for exploratory research and analysis. J Comput Chem. 2004;25(13):1605-12
