# Supplementary material for: Hepatitis B virus core protein phosphorylation: Identification of the SRPK1 target sites and impact of their occupancy on RNA binding and capsid structure
Source: PLoS Pathog. 2018 Dec 19;14(12):e1007488. doi: 10.1371/journal.ppat.1007488 (PMC6317823; doi:10.1371/journal.ppat.1007488)
Supplement: S1 Fig — (PDF) [file ppat.1007488.s003.pdf]

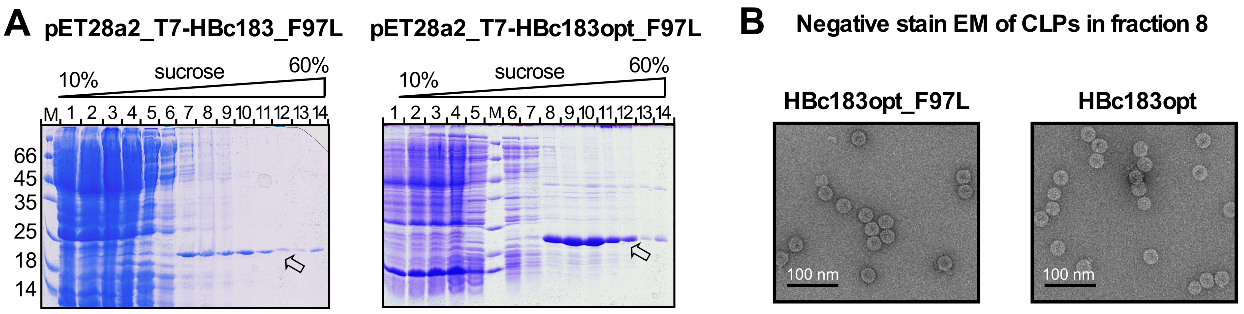

S1 Fig. Increased yield of HBc183_F97L CLPs in *E. coli* by codon usage adaptation. *E. coli* BL21*Cp cells were transformed with pET28a2 vectors carrying the F97L mutation in the context of our conventional HBc183 gene (1) or an *E. coli* codon usage optimized variant (HBc183opt; *this work*). **(A) Sucrose gradient enrichment.** Cleared lysates from the indicated IPTG-induced cultures were sedimented through 10%-60% sucrose gradients as described (2). Fourteen fractions were harvested from the top and analyzed by SDS-PAGE and Coomassie Blue staining. CLPs typically accumulate in fraction 7-11. Yields from the HBc183opt gene were routinely ~3-fold higher. Comparable results were obtained for the wild-type HBc183opt vector. **(B) Negative stain electron microscopy (EM).** Gradient-enriched CLPs from F97L or wild-type HBc183 were stained with uranyl acetate. No F97L-specific differences in particle shape or T=3 vs. T=4 frequency were evident.

Supplementary references:

(1) Nassal M. 1988. Total chemical synthesis of a gene for hepatitis B virus core protein and its functional characterization. Gene 66(2): 279-94

(2) Heger-Stevic J, Kolb P, Walker A, Nassal M. 2018. Displaying whole-chain proteins on hepatitis B virus capsid-like particles. Methods in Molecular Biology 1776:503-531 *In:* Virus-derived Nanoparticles for Advanced Technologies, Eds: Wege & Lomonossoff. DOI: 10.1007/978-1-4939-7808-3_33
