# Supplementary material for: Hepatitis B virus core protein phosphorylation: Identification of the SRPK1 target sites and impact of their occupancy on RNA binding and capsid structure
Source: PLoS Pathog. 2018 Dec 19;14(12):e1007488. doi: 10.1371/journal.ppat.1007488 (PMC6317823; doi:10.1371/journal.ppat.1007488)
Supplement: S3 Fig — (PDF) [file ppat.1007488.s005.pdf]

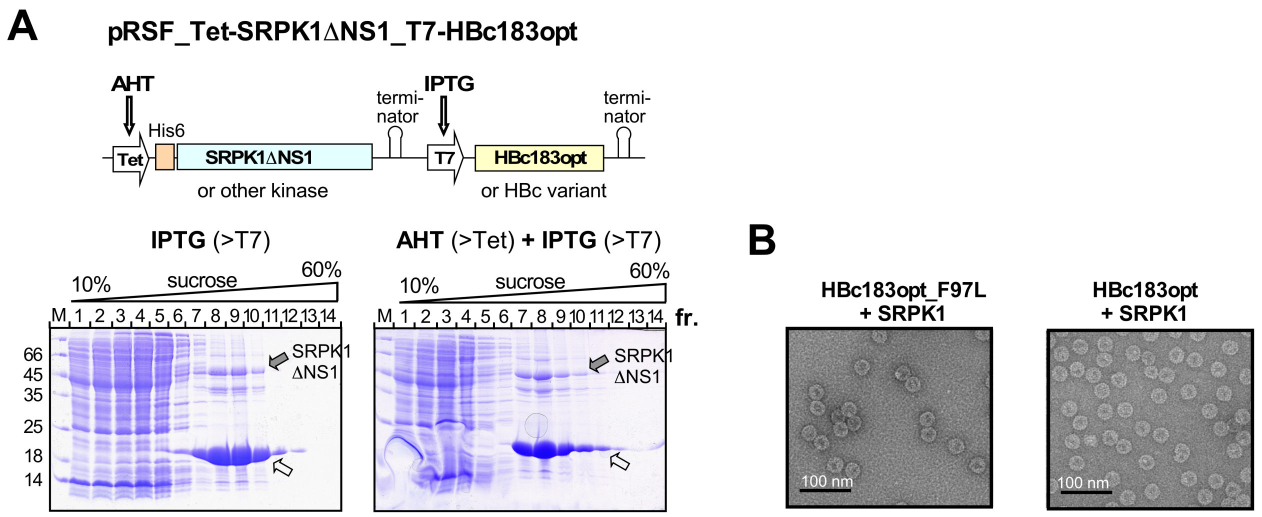

S3 Fig. Efficient coexpression in *E.coli* of HBc183_F97L CLPs with SRPK1. (A) Basic vector design and CLP enrichment via sucrose gradient sedimentation. *E. coli* BL21*Cp were transformed with plasmid pRSF_Tet-SRPK1ΔNS1_T7-HBc183opt_F97L derived from the reporter vector shown in S2 Fig by replacing the two fluorescent protein ORFs by those for SRPK1ΔNS1 and HBc183opt_F97L, respectively (top); other vectors used in this study contained instead ORFs for other kinases and/or other HBc variants. At OD_{600nm} = 0.8 the culture was split and one half was induced using only IPTG (1 mM), the other half using AHT (200 µg/L culture) plus IPTG, as indicated. CLP yields were in either case ~5-fold higher than from the original pET28a2 plasmid (S1 Fig). For SRPK1ΔNS1 we repeatedly saw substantial basal expression without AHT which increased about 3-fold upon AHT addition. Cosedimentation of the kinase with the CLPs (grey and white arrows) is in line with its high affinity for HBc (1). Comparable results were obtained with the respective wild-type HBc183 constructs. **(B) Negative stain EM.** Gradient-enriched CLPs from AHT + IPTG induced cultures coexpressing SRPK1 plus HBc183opt_F97L or wild-type HBc183opt were analyzed using 2% uranyl acetate. No F97L-specific differences were evident.

Supplementary reference to S3 Fig:

(1) Chen C, Wang JC, Zlotnick A. 2011. A kinase chaperones hepatitis B virus capsid assembly and captures capsid dynamics in vitro. PLOS Pathog 7(11): 31002388
