# Supplementary material for: Hepatitis B virus core protein phosphorylation: Identification of the SRPK1 target sites and impact of their occupancy on RNA binding and capsid structure
Source: PLoS Pathog. 2018 Dec 19;14(12):e1007488. doi: 10.1371/journal.ppat.1007488 (PMC6317823; doi:10.1371/journal.ppat.1007488)
Supplement: S4 Fig — (PDF) [file ppat.1007488.s006.pdf]

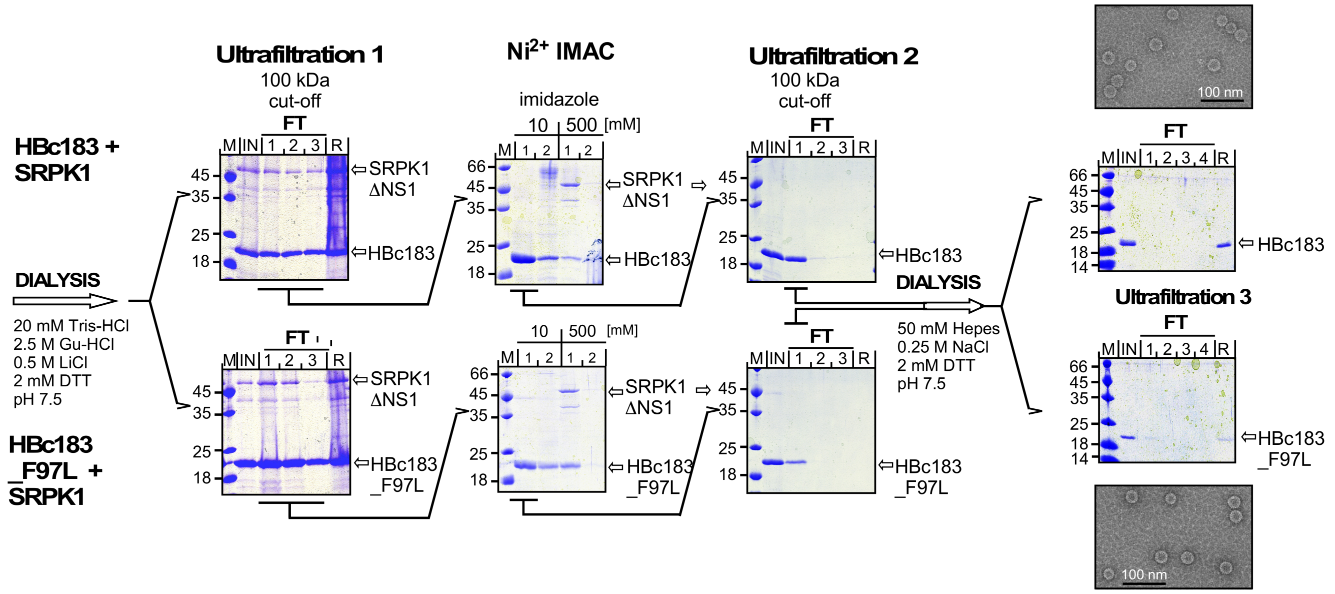

S4 Fig. Separation of HBc183 CLP-associated His6-SRPK1ΔNS1 under semidenaturing conditions. Ni^{2+} IMAC of the gradient fractions containing HBc183 or HBc183_F97L CLPs plus cosedimenting His6-tagged SRPK1ΔNS1 (S3 Fig) failed to separate the kinase, without or with added ATP (1), confirming its high affinity for HBc. To induce SRPK1 dissociation from HBc the samples were dialysed against the indicated 2.5 M guanidium hydrochloride (GuHCl) dissociation buffer (2), then spun through an Amicon ultrafiltration (UF) device (100 kDa cut-off). After 9/10 of the input (IN) had flown through the filter (FT1), the retentate (R) was filled up to the original volume with dissociation buffer and the procedure was repeated twice (yielding FT2, FT3). Then also the retentate was readjusted to the original volume. For GuHCl removal prior to SDS-PAGE, proteins were precipitated with EtOH. Upon the first UF (UF1), most HBc183 as well as SRPK1 were found in FT1 - FT3, indicating successful disassembly. Next, the pooled FT fractions were subjected to Ni^{2+} IMAC in dissociation buffer. The bulk of HBc proteins eluted at 10 mM imidazole while most His6-SRPK1ΔNS1 was only released at 500 mM imidazole, indicating accessibility of the His-tag. Upon UF2, all HBc protein eluted in FT1, confirming its non-assembled state. Dialysis against GuHCl-free high-salt reassembly buffer (2) induced reassembly as shown by retention of the HBc proteins during a final 100 kDa cut-off UF3 and the presence of regular CLPs by negative staining EM. Compared to the in vivo generated CLPs the reassembled HBc183 and HBc183_F97LCLPs both exhibited an increased proportion of ~20% T=3 particles.

Supplementary references to S4 Fig:

(1) Chen C, Wang JC, Zlotnick A. 2011. A kinase chaperones hepatitis B virus capsid assembly and captures capsid dynamics in vitro. PLOS Pathog 7(11): 31002388

(2) Porterfield JZ, Dhason MS, Loeb DD, Nassal M, Stray SJ, Zlotnick A. 2010. Full-length hepatitis B virus core protein packages viral and heterologous RNA with similarly high levels of cooperativity. J Virol 84(14): 7174-84
