# Supplementary material for: Hepatitis B virus core protein phosphorylation: Identification of the SRPK1 target sites and impact of their occupancy on RNA binding and capsid structure
Source: PLoS Pathog. 2018 Dec 19;14(12):e1007488. doi: 10.1371/journal.ppat.1007488 (PMC6317823; doi:10.1371/journal.ppat.1007488)
Supplement: S5 Fig — (PDF) [file ppat.1007488.s007.pdf]

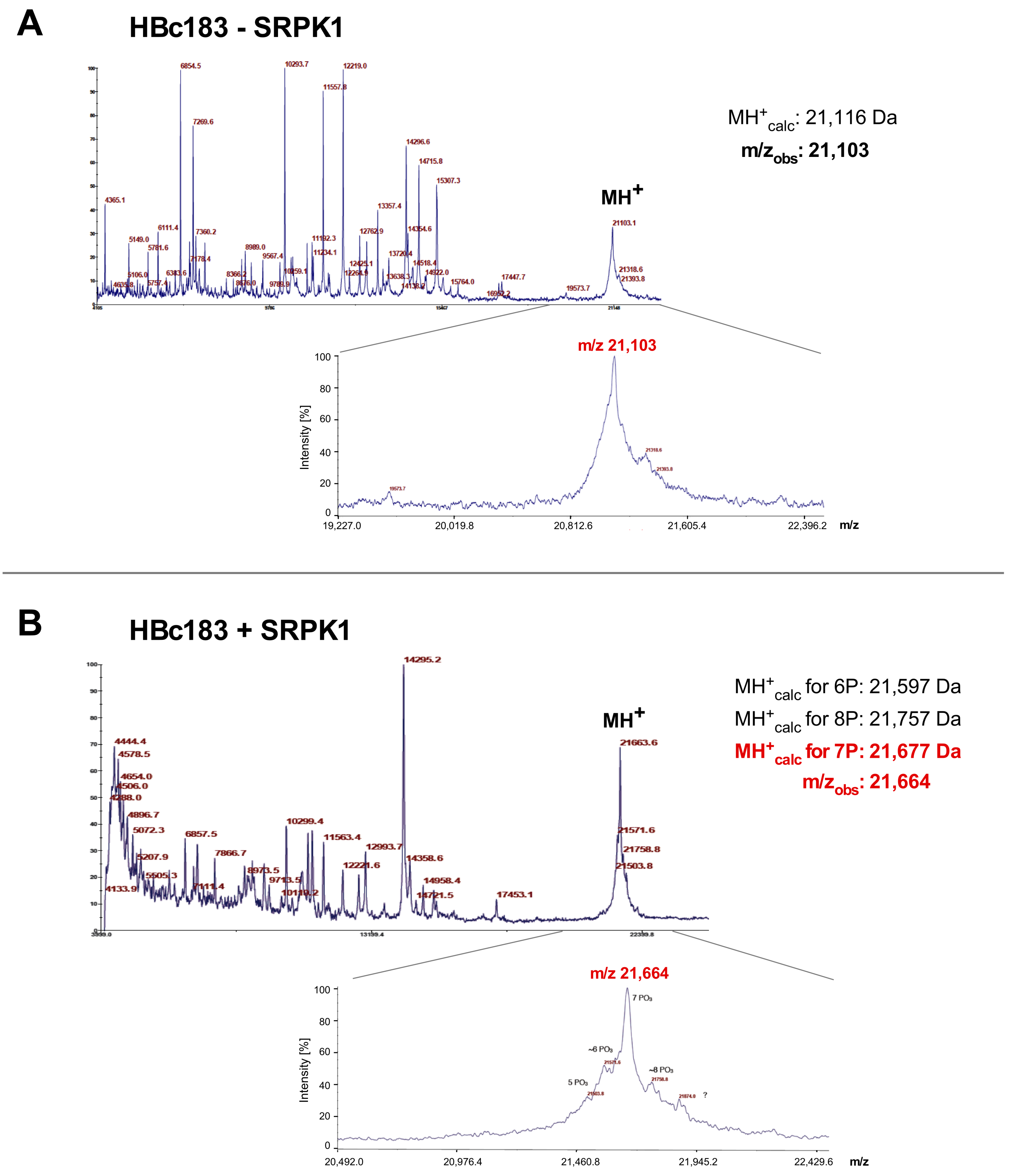

S5 Fig. MALDI-TOF MS characterization of HBc183 expressed in the absence vs. presence of SRPK1ΔNS1. Analyses were performed by Frederic Delolme (UMR5086 CNRS - Université Lyon 1, France) on a Sciex Voyager DE-PRO MALDI-TOF instrument, using saturated sinapic acid as matrix at a sample:matrix dilution of 1:100 (v/v). **(A) HBc183 expressed in the absence of SRPK1.** The observed m/z 21,103 for the dominant MH^+ peak fits best to the calculated MH^+ mass of 21,116 Da for unmodified HBc183 (including the N terminal Met). **(B) HBc183 expressed in the presence of SRPK1.** The observed m/z 21,664 for the dominant MH^+ peak fits best to the calculated MH^+ mass of 21,676 Da for HBc183 carrying 7 phosphoryl groups. While peak intensity is not directly correlated with abundance a rather homogeneous seven-fold phosphorylation status is supported by the uniform appearance of the HBc183 + SRPK1 protein in Phos-Tag SDS-PAGE (Fig 3) and the equal dominance of a seven-fold phosphorylated peptide representing just the CTD (Fig 4).
