# Supplementary material for: Hepatitis B virus core protein phosphorylation: Identification of the SRPK1 target sites and impact of their occupancy on RNA binding and capsid structure
Source: PLoS Pathog. 2018 Dec 19;14(12):e1007488. doi: 10.1371/journal.ppat.1007488 (PMC6317823; doi:10.1371/journal.ppat.1007488)
Supplement: S6 Fig — (PDF) [file ppat.1007488.s008.pdf]

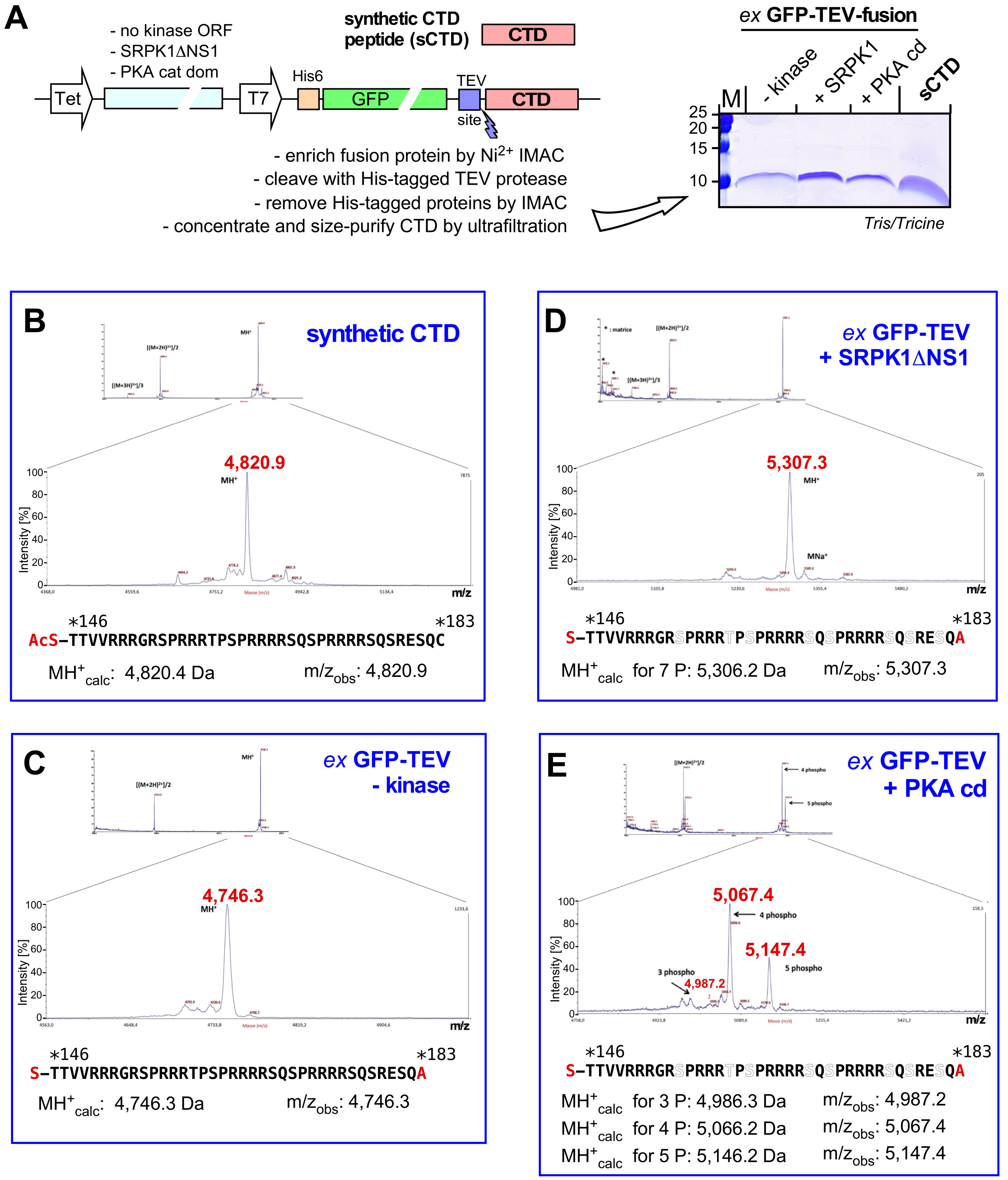

S6 Fig. MALDI-TOF MS analysis of CTD-only peptides confirms seven SRPK1 phosphorylation sites in the HBc CTD and a distinctly lower phosphorylation extent by the catalytic domain of PKA. (A) Scheme of CTD peptide origins. The synthetic CTD (sCTD) was obtained from a commercial supplier (Proteogenix, France). The other CTD peptides were derived by TEV protease clavage from the indicated NHisGFP-TEV-CTD fusions expressed in *E. coli* as such, or coexpressed with SRPK1ΔNS1 or the catalytic domain of cAMP-dependent protein kinase A (PKAcd). Details are provided in S1 Protocols. Final products were analyzed by SDS-PAGE / CBstaining; the gel picture shows a section of the gel in Fig 4C. All CTD peptides routinely co-migrated with the 10 kDa marker; this aberrant mobility is likely due to their very high content of charged amino acids. All samples were then subjected to MALDI-TOF mass spectrometry (B-E). **(B) sCTD. (C) CTD ex fusion protein expressed without kinase. (D) CTD ex fusion protein expressed with SRPK1ΔNS1.** The data in (D) are the same as in Fig 4 and here shown for convenient comparison with the other samples. **(E) CTD ex fusion protein expressed with PKAcd.** In (D) and (E), the potential phosphorylation target residues are shown as outlined fonts.
