# Supplementary material for: Hepatitis B virus core protein phosphorylation: Identification of the SRPK1 target sites and impact of their occupancy on RNA binding and capsid structure
Source: PLoS Pathog. 2018 Dec 19;14(12):e1007488. doi: 10.1371/journal.ppat.1007488 (PMC6317823; doi:10.1371/journal.ppat.1007488)
Supplement: S7 Fig — (PDF) [file ppat.1007488.s009.pdf]

HBc183 wt and non-S/T>A CTD mutants, +/- different kinases

Assembly domain–	*140 *150 *160 *170 *180<br>LSTLPETTVVRRRGRSPRRRTPSPRRRRSQSPRRRRSQSRESQC183	dominant m/z_{obs}	MH+ fits #	P	M_{calc} [Da]
wt	....	21,103	0	P	21,116
wt + SRPK1	....	21,664	7	P	21,676
140	.	15,910	0	P	15,917
140 + SRPK1	.	15,918	0	P	15,917
149	....	16,841	0	P	16,844
149 + SRPK1	....	16,847	0	P	16,844
C183A	....	21,084	0	P	21,082
C183A + SRPK1	....	21,644	7	P	21,656
wt + PKA cd	....	21,361	3	P	21,356
wt + PKC cd	....	21,353	3	P	21,356

HBc183 CTD S/T>A mutants, all + SRPK1

Mutant	Sequence	dominant m/z_{obs}	#	P	M_{calc} [Da]
S155A	...............A............................	21,585	6	P	21,580
T160A	....................A.......................	21,566	6	P	21,561
S162A	......................A.....................	21,579	6	P	21,580
S168A	............................A...............	21,572	6	P	21,580
S170A	..............................A.............	21,576	6	P	21,580
S176A	....................................A.......	21,577	6	P	21,580
S178A	......................................A.....	21,574	6	P	21,580
S181A	....A..	21,659	7	P	21,660
S155A_T160A	...............A....A.......................	21,465	5	P	21,470
S155A_S162A	...............A......A.....................	21,485	5	P	21,484
S162A_S168A	......................A.....A...............	21,483	5	P	21,484
S162*R*_S168A	......................*R*.....A...............	21,569	5	P	21,569
S162A_S168*G*	......................A.....*G*...............	21,467	5	P	21,470
S176A_S178A	....................................A.A.....	21,484	5	P	21,484
S176A_S181A	....................................A....A..	21,560	6	P	21,564
S155A_T160A_S162A	...............A....A.A.....................	21,387	4	P	21,388
T162A_S168*G*_S170A	......................A.....*G*.A.............	21,367	4	P	21,374
S176A_S178A_S181A	....................................A.A..A..	21,464	5	P	21,468

S7 Fig. Predominant number of phosphoryl groups in kinase coexpressed wild-type HBc183 and CTD derivatives by MALDI-TOF MS analysis. The top line shows the C terminal HBc sequence starting at position 140. In the upper block coexpression with a specific kinase is indicated; proteins in the lower block are all HBc183 S/T>A variants coexpressed with SRPK1ΔNS1. Dots indicate identical amino acids. The column "dominant m/z_{obs}" indicates the major observed m/z peak, the next column shows the number of phosphoryl groups giving the closest match to the respective calculated MH^+ mass in Da given in the last column; consider that one phosphoryl group adds ~80 Da. HBc140 and HBc149 coexpressed with SRPK1 showed the same masses as the mono-expressed proteins, indicating a lack of target sites in the assembly domain and the linker. Coexpression with the catalytic domains of PKA and PKC introduced predominantly three phosphoryl groups into the CLP forming wild-type HBc183 protein; for the non-assembling GFP-TEV-CTD fusion protein four- and five-fold phosphorylated species were most abundant (S6 Fig); this may reflect better target site accessibility. For SRPK1, each S/T>A mutation except S181A caused the loss of one phosphoryl group.
