# Supplementary material for: Hepatitis B virus core protein phosphorylation: Identification of the SRPK1 target sites and impact of their occupancy on RNA binding and capsid structure
Source: PLoS Pathog. 2018 Dec 19;14(12):e1007488. doi: 10.1371/journal.ppat.1007488 (PMC6317823; doi:10.1371/journal.ppat.1007488)
Supplement: S8 Fig — (PDF) [file ppat.1007488.s010.pdf]

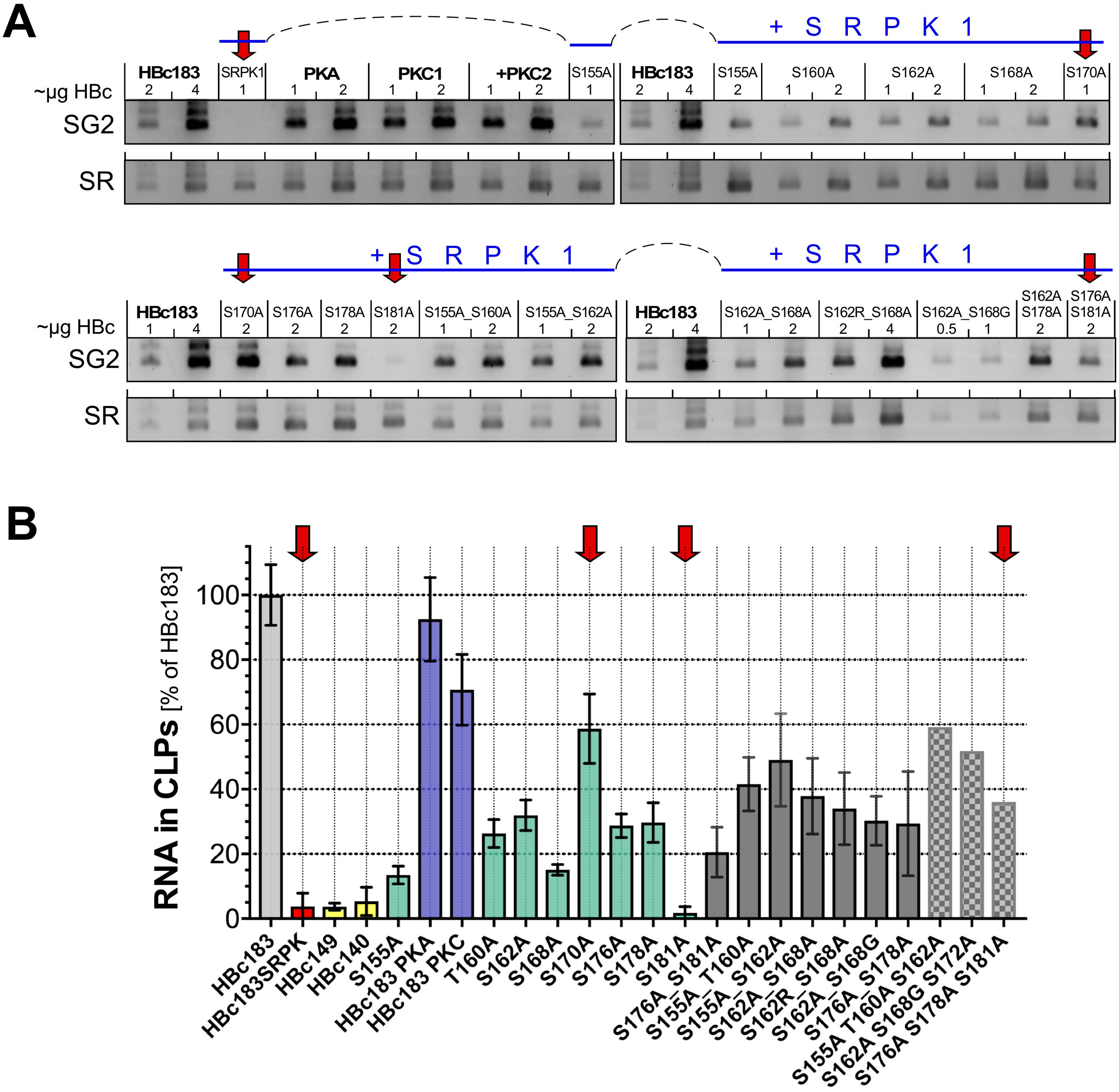

S8 Fig. Differential impact of kinase coexpression and phosphorylation site mutations on CLP RNA content. (A) RNA (SG2) versus protein (SR) staining of CLPs separated by NAGE. Aliquots from the sucrose gradient peak fractions of the respective CLP preparations containing approximately the indicated amounts of HBc protein were subjected to NAGE analysis and post electrophoresis stained with Sybr Green 2 (SG2) for RNA; after recording the SG2 signals (Typhoon Laser scanner) the gels were destained, then restained with Sypro Ruby for protein and the red fluorescence was recorded analogously, using the advanced protocol given in Materials and methods. **(B) Semiquantitative evaluation of CLP RNA content.** The left part of the graph including all single site mutants is as in Fig 5C. The ratio of SG2 vs. SR fluorescence intensity for each band was calculated, and that for HBc183 was set to 100% (n=8). Values for the S/T>A single (aqua) and double (dark grey) variants are derived from ≥3 determinations; error bars represent standard deviation (SD). The triple variants were not systematically analyzed but RNA contents of 50-60% of that in HBc183 CLPs were commonly seen. Red arrows point to variants producing salient values: SRPK1 coexpression reduced RNA content of HBc183 CLPs by >90%, comparable to CTD deletion (HBc149, HBc140); S181A was the only variant with equally low RNA content in line with its wild-type like seven-fold phosphorylation; also, adding S181A to other mutations had little effect (e.g. S176A vs. S176A_S181A; or S176A_S178A_S181A vs. the other triple mutants). For S170A reproducibly higher RNA contents were measured than for the other six-fold phosphorylated single variants, as is already evident by visual comparison of the SG2 vs. SR staining (upper right panel) of S168A CLPs in the neighboring lanes.
