# Supplementary material for: Hepatitis B virus core protein phosphorylation: Identification of the SRPK1 target sites and impact of their occupancy on RNA binding and capsid structure
Source: PLoS Pathog. 2018 Dec 19;14(12):e1007488. doi: 10.1371/journal.ppat.1007488 (PMC6317823; doi:10.1371/journal.ppat.1007488)
Supplement: S9 Fig — (PDF) [file ppat.1007488.s011.pdf]

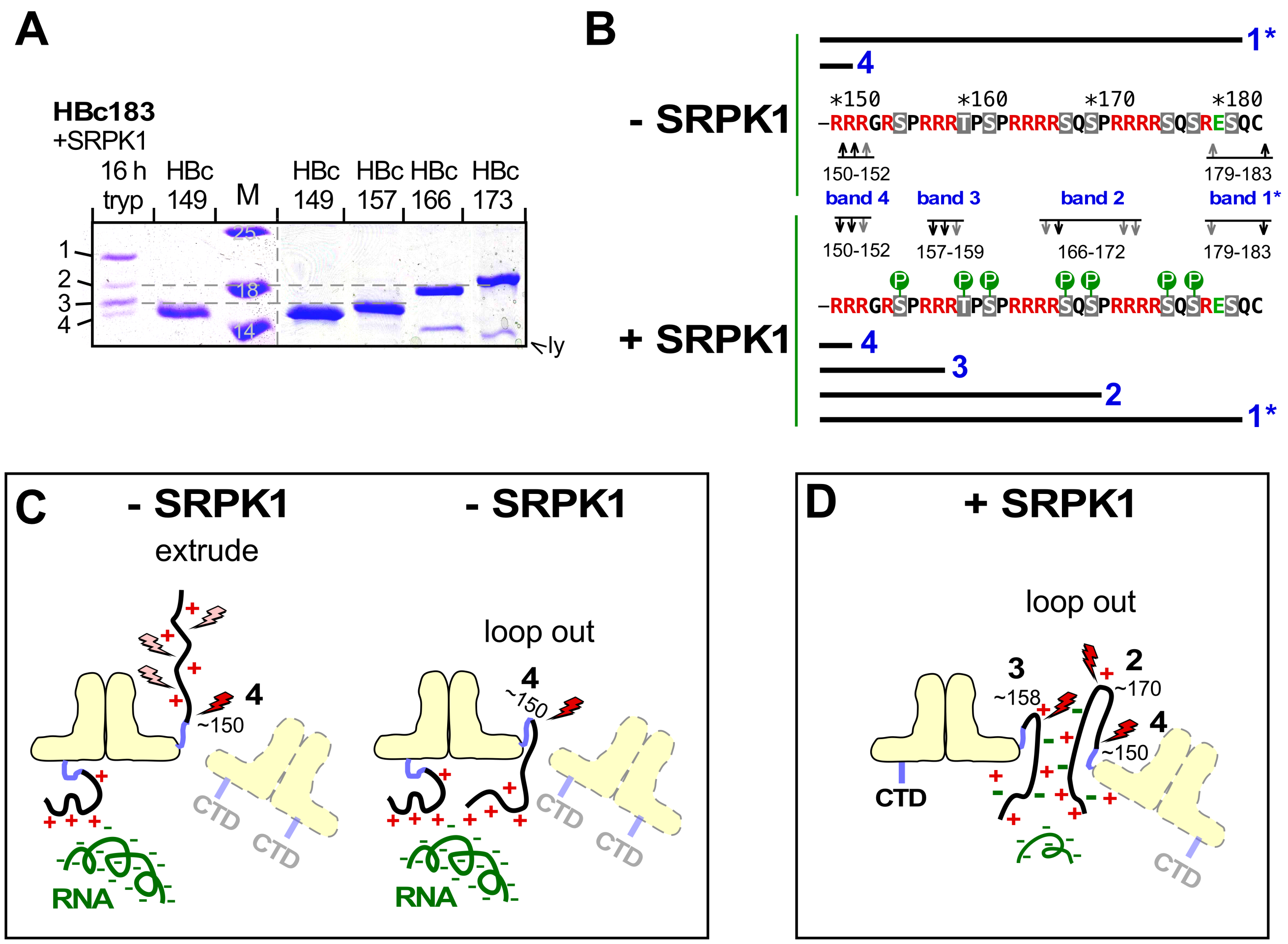

S9 Fig. Schematic models for differential trypsin sensitivity of CTDs in unmodified vs. seven-fold phosphorylated HBc183 CLPs. (A) Approximate mapping of the trypsin cleavage sites in SRPK1-coexpressed HBc183 CLPs by C terminally truncated HBc reference proteins. The indicated protein samples were separated by SDS-PAGE and stained by CB. The gel picture is the same as in Fig 7D, presented here again for convenience. Unmodified HBc183 CLPs showed only two stable bands, namely 1 and 4, upon trypsin treatment (see Fig 7B,C). The 14 kDa band termed "ly" represents lysozyme from the cell lysis procedure. **(B) Linear representation of trypsin cleavage sites in unmodified** ***(top)*** **and seven-fold SRPK1-phosphorylated** ***(bottom)*** **HBc183 CLPs.** The primary sequence of the CTD and the seven phosphorylation sites (green spheres labeled P) are indicated, together with the approximate C termini of bands 1 and 4 (-SRPK1), and 1, 2, 3 and 4 (+SRPK1). The asterisk on the band 1 label indicates that it is unclear whether this band represents fully intact HBc183 or a trypsin processing product cut after R179. **(C) Two models for partial conversion of HBc183 into band 4 in unmodified CLPs.** One option is complete extrusion of a fraction of CTDs, such that after primary cleavage at any site downstream R150 secondary cleavages chew back the CTD until the stably folded N terminal assembly domain is reached. The alternative CTD loop-out model predicts that just the band 4 processing site at around position 150 is accessible to trypsin on a fraction of subunits per CLP. The lack of RNA loss from the partially processed CLPs is more easily envisaged with the loop out model whereby the clipped-off CTDs remain capsid-internally available to neutralize the packaged RNA. **(D) Loop out model for SRPK1-phosphorylated HBc183 CLPs.** For SRPK1-phosphorylated HBc183 CLPs a full CTD extrusion model appears even more unlikely because selective cleavages at the sites generating bands 2, 3 and 4 from entirely exposed, uniformly phosphorylated CTDs is difficult to image. Hence the observed accessibility constraints arise most likely from three-dimensional CLP structure. Electrostatic interactions between the alternating charges in the phospho-CTDs and the inner lining of the capsid lumen might allow specific looping out of the cleavage sites around positions 170 (band 2), 158 (band 3) and 150 (band 4), as schematically depicted; different lengths of C termini sticking out from the capsid surface would also be compatible with the data (not shown). It is tempting to speculate that the proportions of bands 1 to 4 in the different CLPs relate to the four quasi-equivalent types of subunit per T=4 particle. However, owing to the potential presence of non-intact CLPs with only partially closed shells permitting the protease access to *bona fide* internal CTDs this would currently be speculative.
