# Supplementary material for: Hepatitis B virus core protein phosphorylation: Identification of the SRPK1 target sites and impact of their occupancy on RNA binding and capsid structure
Source: PLoS Pathog. 2018 Dec 19;14(12):e1007488. doi: 10.1371/journal.ppat.1007488 (PMC6317823; doi:10.1371/journal.ppat.1007488)
Supplement: S10 Fig — (PDF) [file ppat.1007488.s012.pdf]

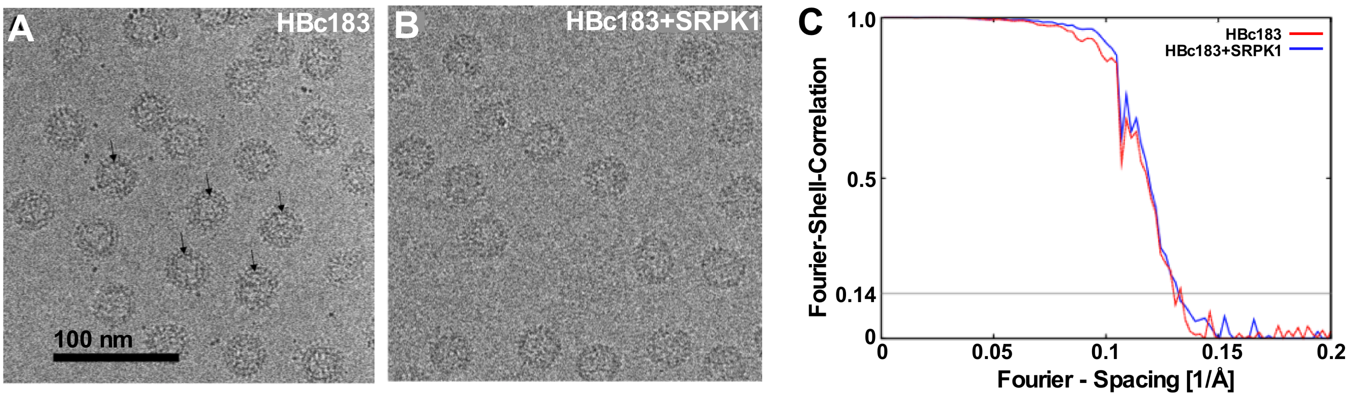

S10 Fig. CryoEM analysis of non-phosphorylated vs. highly phosphorylated HBc183 CLPs. (A,B) Representative micrographs of *E.coli* derived HBc183 CLPs expressed in the absence (A) vs. presence (B) of SRPK1. Images were taken on a Tecnai F20 with a TVIPS F816 CMOS detector. Arrows in (A) mark a ring of internal density that is absent from the SRPK1-coexpressed particles. Equatorial slices from the respective image reconstructions are shown in Fig 8B. **(C) Fourier-Shell-Correlation.** The graph shows the Fourier-Shell-Correlations for non-phosphorylated (red) vs. highly phosphorylated CLPs (blue) after gold standard refinement of half data sets. Accordingly, nominal resolution of both reconstructions was just below 8 Å.
