# Supplementary material for: Hepatitis B virus core protein phosphorylation: Identification of the SRPK1 target sites and impact of their occupancy on RNA binding and capsid structure
Source: PLoS Pathog. 2018 Dec 19;14(12):e1007488. doi: 10.1371/journal.ppat.1007488 (PMC6317823; doi:10.1371/journal.ppat.1007488)
Supplement: S2 Fig — (PDF) [file ppat.1007488.s004.pdf]

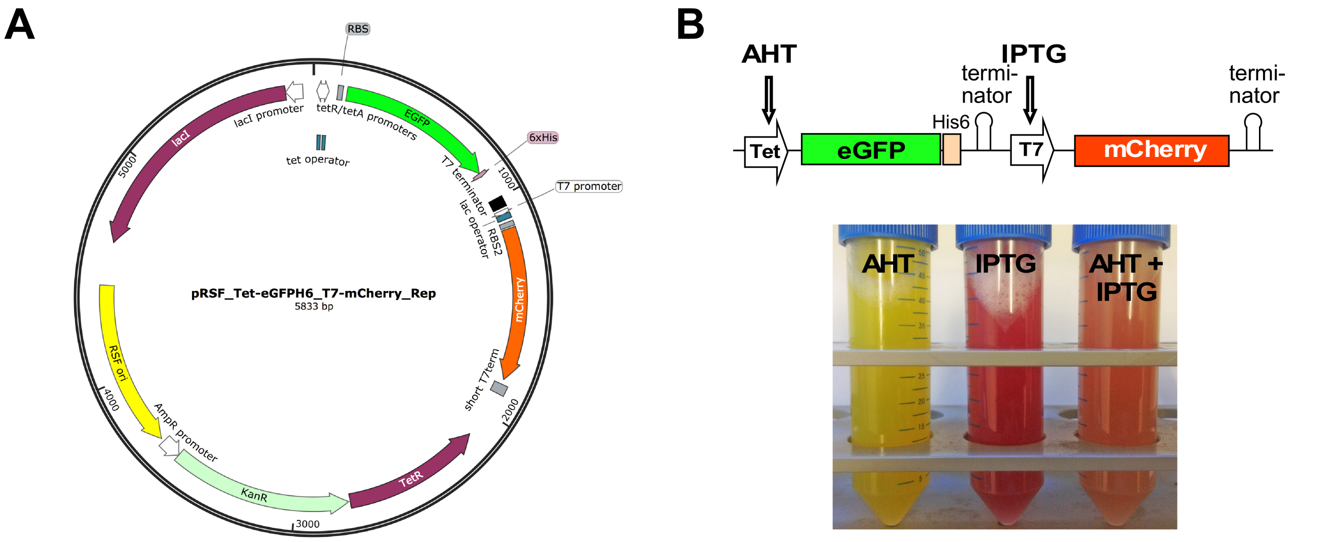

S2 Fig. Vector system for regulated coexpression of two gene products in *E. coli*. (A) Complete map of prototypical dual reporter vector pRSF_Tet-eGFP_T7-mCherry_Rep. Pertinent vector features are highlighted on this model plasmid encoding C-terminally His6-tagged eGFP under control of the Tet promoter which via the encoded Tet repressor (TetR) is inducible by anhydrotetracycline (AHT); and mCherry under control of the T7 promoter whereby expression of T7 RNA polymerase in BL21 *E. coli* strains is inducible, via the encoded lac repressor (Lac I), by isopropyl β-1-D-thiogalactopyranoside (IPTG). RBS, ribosome binding site; T7term, T7 terminator. **(B) Inducer-specific expression of eGFP vs. mCherry.** The scheme is a cutout from (A) emphasizing the option of separate induction of the Tet and T7 promoters by AHT and IPTG. Cultures of reporter vector-transformed *E.coli* BL21*Codonplus cells were induced at 25°C for 14 h with either AHT, or IPTG, or both. The different colors of the cultures are in line with the expected promoter selectivities. However, expression levels are also affected by the specific gene sequences and protein-chemical properties of their products; hence the total amounts as well as the ratio of the steady-state levels can substantially differ for different gene combinations.
