# Supplementary material for: Hepatitis B virus core protein phosphorylation: Identification of the SRPK1 target sites and impact of their occupancy on RNA binding and capsid structure
Source: PLoS Pathog. 2018 Dec 19;14(12):e1007488. doi: 10.1371/journal.ppat.1007488 (PMC6317823; doi:10.1371/journal.ppat.1007488)
Supplement: S12 Fig — (PDF) [file ppat.1007488.s014.pdf]

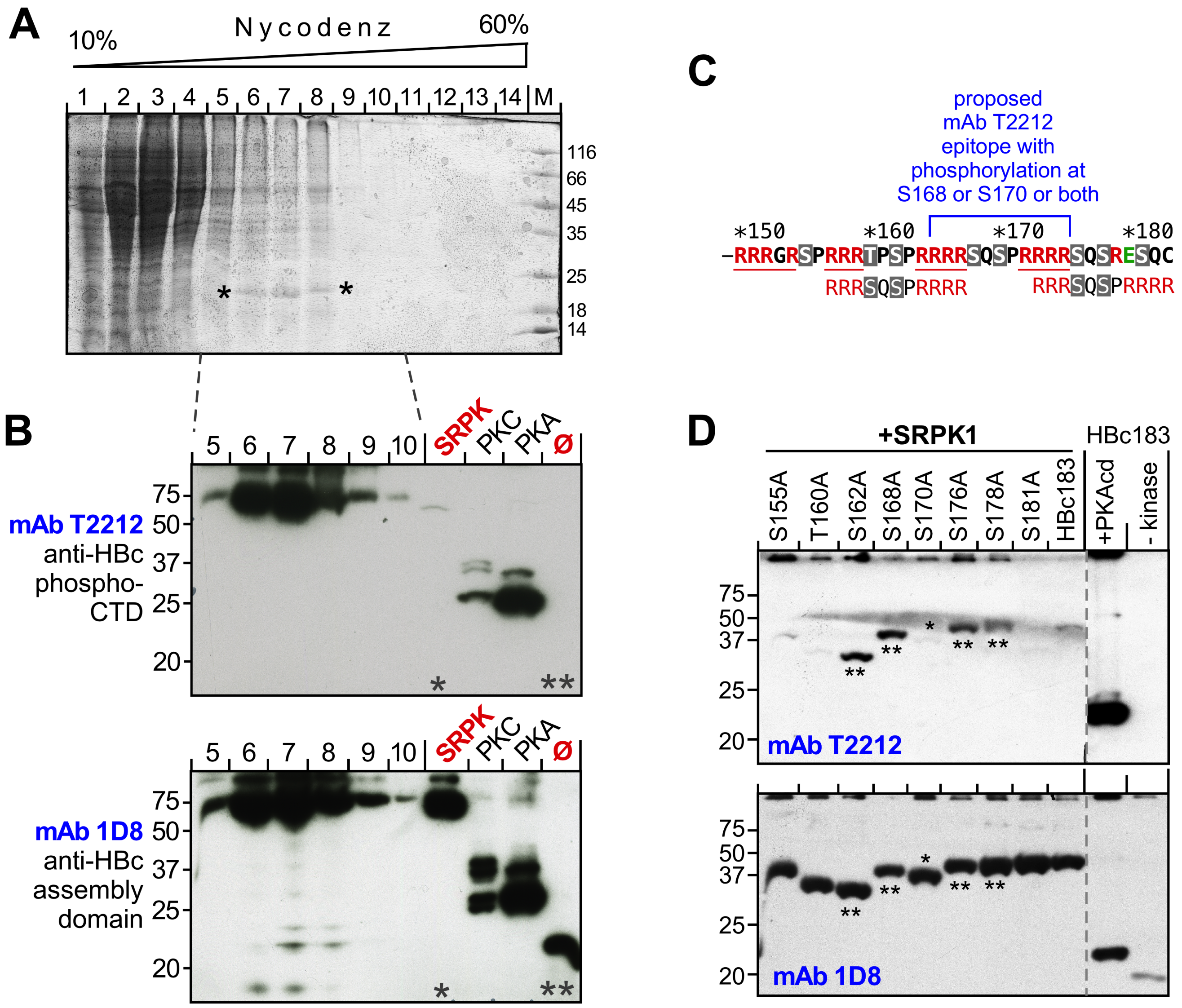

S12 Fig. Similarly high though non-identical phosphorylation of HBc183 in human cells vs. recombinant SRPK1-phosphorylated HBc183. (A) Enrichment of intracellular HBV capsids from induced HepG2.117 cells. The CB stained SDS-PAGE gel from the Nycodenz gradient is the same as in Fig. 9 except the entire gel is shown. Asterisks mark a band likely representing HBc183. **(B) Differential recognition by phospho-CTD specific mAb T2212 of differently phosphorylated HBc183.** The indicated Nycodenz gradient fractions and recombinant HBc proteins were separated by Phos-Tag SDS-PAGE and immunoblotted using mAb T2212 which reportedly recognizes a phosporylation-dependent epitope between positions 165 and 175 in the CTD (1). The mAb reacted well with the major, strongly retarded HBc from the hepatoma cells (at ~75 kDa), and also with the main product of PKA-coexpressed HBc183. The PKC coexpression products reacted weakly whereas no signals were detected for HBc183 coexpressed with SRPK1 or without kinase; their presence on the blot was shown by the mAb 1D8 blot (lanes *, **) which is the same as in Fig. 9 **(C) Proposed mAb T2212 epitope.** The immunogen were capsids from HBV-positive patient serum; cognate antigen binding could be blocked by a peptide corresponding to HBc aa 165-175 but only when phosphorylated with “bovine heart kinase”, i.e. PKA (Machida et al. 1991). It was concluded that the epitope comprises aa 165-175 with phosphorylated S168 or S170 or both. Notably, due to its repetitive nature the CTD contains additional (partially) similar sequence motifs as shown by the alignments below the CTD sequence. **(D) Mutating individual SRPK1 phosphorylation sites within and adjacent to the proposed T2212 epitope but not at S170 restores recognition by mAb T2212.** Recombinant HBc183 and the indicated S/T>A variants coexpressed with SRPK1 plus wt HBc183 coexpressed with the catalytic domain of PKA or without kinase were separated by Phos-Tag SDS-PAGE, then immunoblotted using mAb T2212 and reprobed using mAb 1D8 as loading control. MAb T2212 reacted intensely with PKA-coexpressed but only marginally with SRPK1-coexpressed HBc183. However, mutations S162A and S168A and, less pronouncedly, S176A and S178A (marked by **) but not S170A (marked by *) restored reactivity. This is in line with mAb T2212 binding requiring phosphorylated S170 yet also a close-by unmodified hydroxy amino acid side-chain. More detailed mapping will require further experiments, but the current data indicate that despite similar Phos-Tag retardation the bulk of mammalian intracellular HBc and seven-fold SRPK1-phosphorylated recombinant HBc183 differ in at least one feature that is relevant for T2212 recognition.

Supplementary reference to S12 Fig:

(1) Machida A, Ohnuma H, Tsuda F, Yoshikawa A, Hoshi Y et al. Phosphorylation in the carboxyl-terminal domain of the capsid protein of hepatitis B virus: evaluation with a monoclonal antibody. J Virol 1991; 65(11):6024-30
