# Supplementary material for: Hepatitis B virus core protein phosphorylation: Identification of the SRPK1 target sites and impact of their occupancy on RNA binding and capsid structure
Source: PLoS Pathog. 2018 Dec 19;14(12):e1007488. doi: 10.1371/journal.ppat.1007488 (PMC6317823; doi:10.1371/journal.ppat.1007488)
Supplement: S11 Fig — (PDF) [file ppat.1007488.s013.pdf]

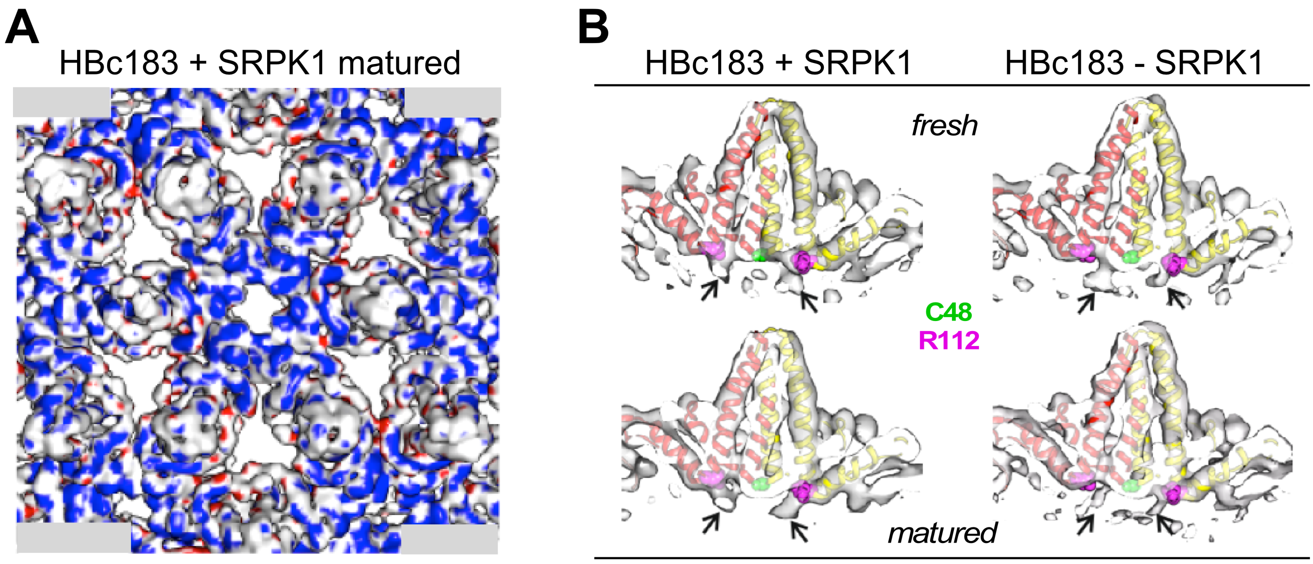

S11 Fig. Long-term storage induces a more ordered structure especially in SRPK1-phosphorylated HBc183 CLPs. (A) Electron density differences between matured and fresh CLPs. The surface representation shows a close-up of a two-fold symmetry axis from a reconstruction at nominally 6.6 Å resolution of SRPK1-coexpressed HBc183 CLPs, stored at 4°C for 1.5 years before vitrification. The major differences between these matured and fresh CLPs (Fig 8C) are highlighted in color; blue indicates more density in the reconstruction of the fresh CLPs, red indicates the reverse. The accumulation of red color at the local three-fold axes indicates increased ordering of the N termini which embrace the spikes. Differences between fresh and matured non-phosphorylated CLPs were much less pronounced. **(B) Internal electron density remains in contact with R112 upon maturation.** The crystal structure of the HBc CD dimer in HBc149 CLPs (pdb: 1QGT) was modeled into the density maps of fresh and matured SRPK1-phosphorylated (left) or non-phosphorylated CLPs (right), leaving in all cases unaccounted for internal density (grey). Especially in the SRPK1-coexpressed CLPs this extra density (arrows) contacted R112 (magenta spheres), near C48 (green spheres); these contacts remained during maturation. Given the low RNA content of the phosphorylated CLPs, the extra density must largely derive from the CTD. Similarly localized but less prominent extra density was also seen in fresh and matured non-phosphorylated HBc183 CLPs; however, due to their high RNA content a clear distinction between nucleic acid and CTD residues is difficult.
